# Genome‐wide screen reveals a universal role of ATP in ciprofloxacin tolerance among genetically distinct *Escherichia coli* persisters

**DOI:** 10.1002/mlf2.70072

**Published:** 2026-03-19

**Authors:** Zhenfang Mei, Yawen Cai, Jianfeng Huang, Kedong Zhao, Zuqin Zhang, Dandan Yu, Shiyan Lu, Zeying Lai, Thomas K. Wood, Xinmiao Fu

**Affiliations:** ^1^ Provincial University Key Laboratory of Cellular Stress Response and Metabolic Regulation, College of Life Sciences Fujian Normal University Fuzhou China; ^2^ Institute of Precision Medicine, Fujian Provincial Hospital Fuzhou University Affiliated Provincial Hospital Fuzhou China; ^3^ Department of Chemical Engineering Pennsylvania State University University Park Pennsylvania USA

**Keywords:** antibiotic uptake, ATP, ciprofloxacin, persister, ROS

## Abstract

Bacterial persisters show tolerance to bactericidal antibiotics and play essential roles in chronic infections; however, the general mechanisms underlying persister formation and antibiotic tolerance remain insufficiently characterized. In this study, the *Escherichia coli* Keio library was used to identify genes involved in ciprofloxacin tolerance by culturing each mutant to the late stationary phase (to induce persistence via starvation), followed by dilution into fresh medium for antibiotic exposure. This two‐step, genome‐wide screening approach enabled the identification of 37 ciprofloxacin‐sensitive mutants with diverse biological functions and 11 ciprofloxacin‐tolerant mutants related to amino acid and β‐nicotinamide adenine dinucleotide (NAD⁺) biosynthesis, with 25 genes being identified as persister‐related genes for the first time. Notably, sensitive mutants (Δ*atpC*, Δ*atpF*, Δ*ruvC*, and Δ*rnr*) were specifically sensitive to quinolone antibiotics, whereas tolerant mutants (Δ*metR*, Δ*leuB*, and Δ*nadB*) showed tolerance to ampicillin and gentamicin. Importantly, adenosine triphosphate (ATP) levels were downregulated in ciprofloxacin‐tolerant mutants and upregulated in ciprofloxacin‐sensitive mutants, implying a negative correlation between ATP levels and ciprofloxacin tolerance among these genetically distinct persisters. This negative correlation was further observed when ATP levels in different mutants were chemically modulated using specific metabolites, nutrients, and respiration inhibitors. In addition, ciprofloxacin persistence across different mutants was found to correlate closely with antibiotic uptake and reactive oxygen species (ROS) levels. Collectively, these findings establish a universal role for ATP in the ciprofloxacin tolerance of genetically diverse persisters under varying resuscitation conditions, conceivably through the modulation of antibiotic uptake and ROS accumulation, and it is implied that the provision of abundant nutrients is potentially beneficial for anti‐persister chemotherapy in clinic settings.

## Impact statement

This research demonstrates that diverse gene mutations modulate the survival of bacterial persister cells, a major contributor to recurrent and chronic bacterial infections, under ciprofloxacin treatment. Nevertheless, the critical determinant of ciprofloxacin tolerance among genetically distinct persisters is generally associated with cellular energy (i.e., ATP) levels: lower ATP levels are associated with increased ciprofloxacin tolerance, whereas ATP elevation through the provision of abundant nutrients enhances ciprofloxacin efficacy against persisters. This study not only provides novel insights into the mechanisms underlying ciprofloxacin tolerance in bacterial persisters but also establishes a foundation for the development of nutritional interventions as a potential strategy for the treatment of recurrent and chronic infections.

## INTRODUCTION

Bacterial infection remains the second leading cause of human mortality, accounting for more than 7.7 million deaths in 2019^1^. Persisters are non‐growing or slow‐growing phenotypic variants of bacteria that are able to survive treatment with bactericidal antibiotics or other stressful environments^2^. Although persisters typically constitute only a small fraction of the bacterial population (usually less than 1%), they play critical roles in chronic and persistent infections^3^, ^4^, ^5^, contribute to the failure of antibiotic therapy^6^, ^7^, facilitate the evolution of antibiotic resistance^8^, ^9^, which has become a global threat to human health^10^, ^11^, and undermine host immune defenses^12^. Since persisters were first described in the 1940s^13^, ^14^, the molecular mechanisms underlying their formation and antibiotic tolerance have been extensively investigated, particularly in recent decades. Multiple biological pathways have been implicated in persister formation and antibiotic tolerance, including toxin–antitoxin systems, DNA repair systems, quorum sensing, the stringent response, bacterial motility, biofilm formation, and immune evasion^15^. Nevertheless, the general molecular mechanisms governing persister formation and the precise contributions of individual biological pathways remain poorly defined^15^, ^16^.

One major obstacle in persister research is the extremely low proportion of persisters within bacterial populations, which limits the direct application of conventional approaches such as transcriptomic, proteomic, and metabolomic analyses unless persisters are substantially enriched or induced^15^. Another challenge arises from the dependence of persister formation not only on genetic background and cell density but also on environmental variables, including pH, oxygen availability, nutrient conditions, and various stressors^16^. In particular, persisters are strongly influenced by experimental conditions, as the duration, concentration, and type of antibiotic exposure are critical determinants^17^, ^18^, ^19^. To provide conceptual clarity, Balaban and colleagues proposed that bacterial persisters can be categorized into two classes: type I persisters, which are induced by stress signals such as starvation, and type II persisters, which arise spontaneously during steady‐state exponential growth in the absence of apparent stress^20^.

Given the distinct characteristics of type I and type II persisters, specific experimental strategies and models are required to dissect their respective formation mechanisms and antibiotic tolerance. Historically, the genetic basis of persister formation has been extensively investigated using diverse genomic libraries in mixed‐ or single‐gene arrayed formats, including the *Escherichia coli* Keio library^21^, ^22^, ^23^, ^24^, ^25^, ^26^, ^27^, ^28^ and the ASKA library^27^, ^29^, ^30^, ^31^, ^32^, transposon libraries of *E. coli* and *Pseudomonas aeruginosa* ^33^, ^34^, ^35^, ^36^, ^37^, ^38^, as well as a *Mycobacterium tuberculosis* CRISPRi library^39^. Meanwhile, screening conditions have varied considerably: antibiotics have been applied to mixed genomic libraries in the stationary phase to eliminate non‐persisters, thereby enriching persisters for isolation^21^, ^22^, ^23^, ^28^, ^34^, ^40^; antibiotics have been administered to exponential‐phase cultures^36^, ^38^, ^41^; or exponential‐ or stationary‐phase cells have been directly spotted onto antibiotic‐containing Luria broth (LB) agar plates^24^, ^25^, ^31^, ^35^, ^37^, ^42^. Despite this methodological diversity, these studies have collectively identified a broad spectrum of genes and pathways implicated in bacterial persistence^15^.

In this study, starvation‐induced type I persisters were generated by culturing each clone of the *E. coli* Keio library to the late stationary phase, followed by dilution into fresh medium containing antibiotics. Under these conditions, both antibiotic‐susceptible cells and antibiotic‐tolerant cells (i.e., persister cells) are eliminated upon resuscitation in fresh medium, as previously reported^19^, ^43^. Consequently, time‐dependent killing of persisters is expected to reflect bacterial dormancy depth^44^, which determines the lag time for persister regrowth^45^, ^46^ and may therefore facilitate the identification of novel genes and pathways involved in persister antibiotic tolerance. Using this approach, 37 ciprofloxacin‐sensitive mutants and 11 ciprofloxacin‐tolerant mutants were identified, 25 of which have not been previously reported. Importantly, although these genetically distinct mutants use functionally diverse pathways to mediate ciprofloxacin tolerance, adenosine triphosphate (ATP) levels were identified as a universal determinant of ciprofloxacin tolerance. These findings advance the understanding of a shared mechanism governing persister resuscitation and highlight the therapeutic potential of nutritional interventions to potentiate anti‐persister chemotherapy.

## RESULTS

### Genome‐wide screen reveals multifaceted biological pathways involved in *E. coli* ciprofloxacin tolerance

**Figure 1:**
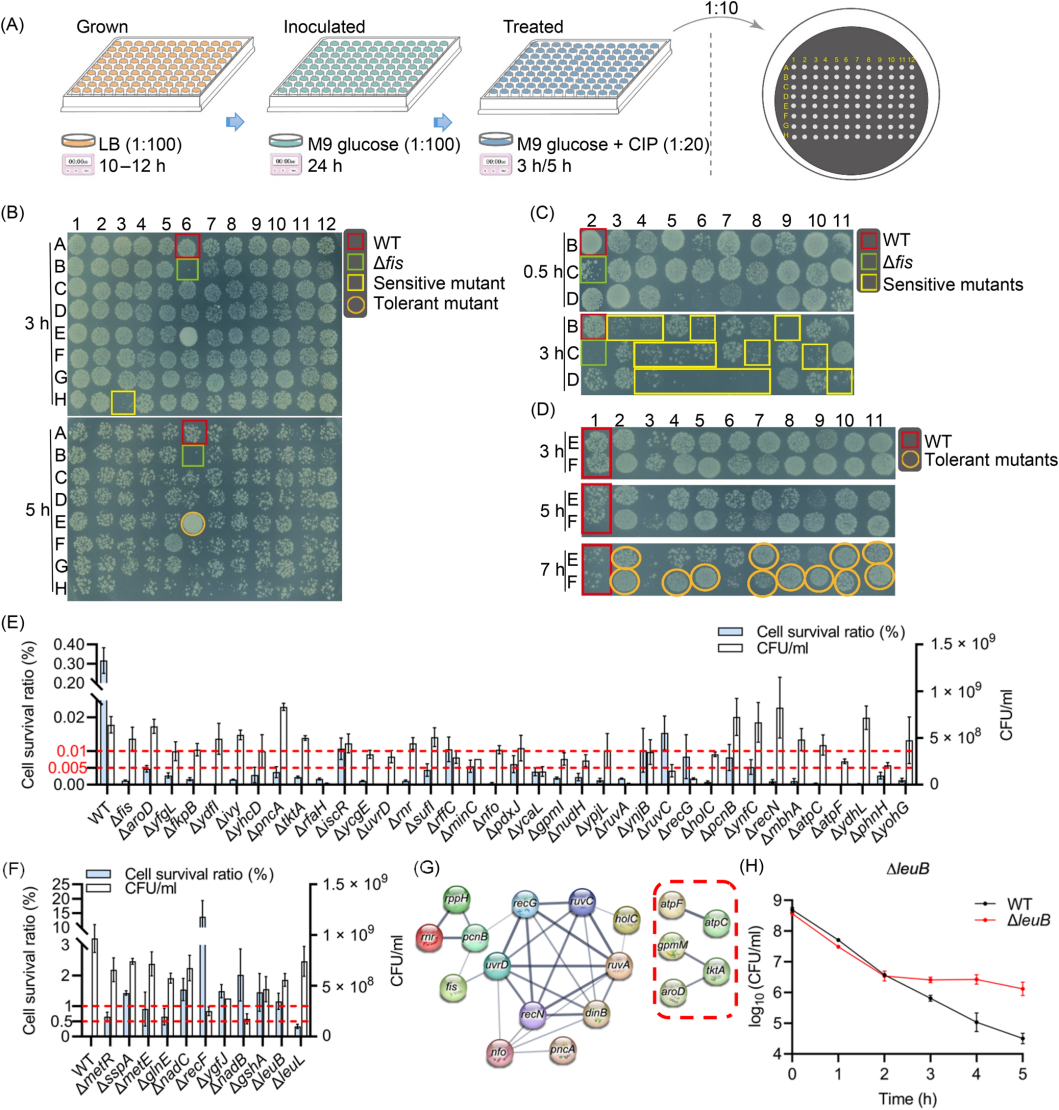
Screening of ciprofloxacin‐tolerant mutants from the Keio library. (A) Schematic diagram of the genome‐wide screening strategy. Individual members of the complete Keio collection (3985 strains) were cultured in Luria broth (LB) medium for 10–12 h, transferred to M9 glucose medium and incubated for 24 h to induce persistence via starvation, diluted 20‐fold into fresh M9 glucose medium containing 2.5 μg/ml ciprofloxacin (CIP), and further incubated for 3 or 5 h for antibiotic killing. Aliquots (4 μl) of 10‐fold diluted cultures were subsequently spotted onto LB agar plates for cell survival assays. (B) Survival of mutants from the Keio collection following the initial screen described in panel A. A representative 96‐well plate is shown. The numbers represent the column, and the letters represent the row; results from the complete initial screen are presented in Figure S2. (C, D) Survival of ciprofloxacin‐sensitive (C) and ciprofloxacin‐tolerant (D) mutants identified in the initial screen and subjected to re‐screening to verify their ciprofloxacin tolerance phenotypes. Representative results are shown, the numbers represent the column, and the letters represent the row. The complete re‐screening results are presented in Figure S3. (E, F) Cell survival ratios of ciprofloxacin‐sensitive (E) and ciprofloxacin‐tolerant (F) mutants following 3 and 5 h of ciprofloxacin treatment, respectively. Absolute CFU values are shown on the right *y*‐axis. Survival ratios were calculated as the ratio of CFU after antibiotic treatment to CFU before treatment. (G) Protein–protein interaction network of ciprofloxacin‐sensitive mutants generated using the STRING database. Genes associated with energy metabolism are highlighted by red dash frame. (H) Time‐dependent killing curves of the wild‐type strain and the Δ*leuB* mutant following treatment with 2.5 μg/ml ciprofloxacin during resuscitation. Results for other ciprofloxacin‐tolerant mutants are shown in Figure S5B.

Given the complexity of persister formation mechanisms^15^, ^16^, ^17^, ^47^, a genome‐wide approach was undertaken to identify genetic determinants of antibiotic tolerance in persisters formed under starvation conditions and subsequently eliminated during resuscitation under nutrient‐rich conditions. To this end, persisters were induced by culturing each mutant from the *E. coli* Keio library, comprising nearly 4000 single‐gene deletion mutants^48^, in 96‐well plates containing M9 glucose medium until the late stationary phase. These persisters were then subjected to killing during resuscitation following a 20‐fold dilution into fresh medium containing ciprofloxacin, as illustrated in Figure 1A.

During optimization of experimental conditions, time‐course assays enabled the identification of sensitive mutants after 1 or 3 h of antibiotic exposure and tolerant mutants after 5 or 7 h (indicated by blue and red text in Figure S1, respectively). Accordingly, time points of 3 and 5 h were selected for the identification of sensitive and tolerant mutants, respectively. In addition, antibiotic dosage assays demonstrated that ciprofloxacin concentrations ranging from 0.625 to 5.0 μg/ml effectively eliminated both sensitive and tolerant mutants (Figure S1). Consequently, 2.5 μg/ml ciprofloxacin was selected for screening, in part based on previous reports^3^, ^49^. Results from the initial Keio library screen are presented in Figure S2, with representative examples shown in Figure 1B.

In the initial genome‐wide screen, 150 sensitive mutants and 20 tolerant mutants were identified. These candidates were subsequently re‐screened under identical conditions for varying durations (Figures S3 and 1C,D). Ultimately, 37 sensitive mutants and 11 tolerant mutants were confirmed (Table 1), and their ciprofloxacin tolerance levels were quantified (right column of Table 1). Specifically, the wild‐type strain showed a survival ratio (i.e., persister proportion) of approximately 0.31% following 3 h of ciprofloxacin exposure, whereas survival rates of sensitive mutants ranged from 0.001% to 0.02% (Figure 1E). After 5 h of ciprofloxacin treatment, the survival rate of the wild type decreased to 0.0047%, while tolerant mutants displayed survival rates ranging from 0.33% to 15% (Figure 1F). Notably, most of the 37 ciprofloxacin‐sensitive mutants and 11 ciprofloxacin‐tolerant mutants retained their respective sensitivity or tolerance profiles when treated with 0.625 μg/ml ciprofloxacin (Figure S4B) or with 2.5 μg/ml ofloxacin, another commonly used quinolone antibiotic (Figure S4C).

**Table 1:** Identified ciprofloxacin persistence genes and their functional classification.

No.	Knockout strain	Annotation	Functional classification	Tolerance level	Newly discovered
Sensitive mutants					
1	Δ*fis*	DNA‐binding protein	Regulatory supercoil	−−a	
2	Δ*rnr*	Ribonuclease R	mRNA catabolic process	−−−	*^b^
3	Δ*uvrD*	DNA helicase II	DNA replication/repair	−−−	
4	Δ*nfo*	Endonuclease IV	DNA repair	−−−	*
5	Δ*ruvA*	Holliday junction ATP‐dependent DNA helicase	DNA replication/repair	−−−	
6	Δ*ruvC*	Crossover junction endodeoxyribonuclease	DNA repair	−	
7	Δ*holC*	DNA polymerase III subunit χ	DNA replication	−−−	*
8	Δ*recG*	ATP‐dependent DNA helicase	DNA repair	−−	
9	Δ*recN*	DNA repair protein	DNA repair	−−−	
10	Δ*atpC*	ATP synthase F1 complex subunit ε	Energy	−−−−	*
11	Δ*atpF*	ATP synthase Fo complex subunit b	Energy	−−−	
12	Δ*yfgL*	Outer membrane protein assembly factor BamB	Cell outer membrane	−−−	*
13	Δ*rfaH*	Transcription antitermination protein RfaH	Transcription regulation	−−−	*
14	Δ*pdxJ*	Pyridoxine 5′‐phosphate synthase	Synthesis of vitamin B6	−−	*
15	Δ*gpmI*	2,3‐bisphosphoglycerate‐independent phosphoglycerate mutase	Glycolysis	−−−	*
16	Δ*pncA*	Nicotinamidase	Pyridine nucleotide biosynthesis	−−−	
17	Δ*tktA*	Transketolase 1	Pentose phosphate pathway	−−−	
18	Δ*rffC*	dTDP‐fucosamine acetyltransferase	Enterobacterial common antigen biosynthetic	−	*
19	Δ*aroD*	3‐dehydroquinate dehydratase	Chorismate biosynthesis	−−−	*
20	Δ*iscR*	HTH‐type transcriptional regulator	Regulates Fe‐S cluster biogenesis	−	
21	Δ*ycgE*	HTH‐type transcriptional repressor BluR	Biofilm maturation	−−−	
22	Δ*minC*	Septum site‐determining protein MinC	Cell division	−−	*
23	Δ*sufI*	Cell division protein FtsP	Cell division	−−−	
24	Δ*nudH*	RNA pyrophosphohydrolase	mRNA decay	−−−	
25	Δ*ivy*	Inhibitor of vertebrate lysozyme	lysozyme inhibitor	−−−	*
26	Δ*phnH*	Alpha‐d‐ribose 1‐methylphosphonate 5‐triphosphate synthase subunit PhnH	Organic phosphonate transport	−−−	*
27	Δ*yohG*	Putative multidrug resistance outer membrane protein MdtQ	Transmembrane transport	−−−	
28	Δ*ycaL*	Metalloprotease YcaL	Proteolysis involved in the protein catabolic process	−−−	*
29	Δ*ypjL*	Predicted inner membrane protein	Arsenite transmembrane transporter activity	−−−	*
30	Δ*ydfI*	Uncharacterized oxidoreductase YdfI	Mannitol metabolic process	−−−	
31	Δ*ydhL*	Uncharacterized protein YdhL	Response to hydrogen peroxide	−−−	
32	Δ*fkpB*	FKBP‐type 16 kDa peptidyl‐prolyl cis‐trans isomerase	Chaperone‐mediated protein folding	−−−	
33	Δ*pcnB*	Poly(A) polymerase I	RNA modification	−−	*
34	Δ*ynfC*	UPF0257 lipoprotein YnfC	Plasma membrane	−−	*
35	Δ*ynjB*	Uncharacterized protein YnjB	Transmembrane transport	−	*
36	Δ*yhcD*	Uncharacterized outer membrane usher protein YhcD	Pilus assembly	−−−	
37	Δ*mbhA*	Putative truncated flagellar export/assembly protein LafU	‐	−−−	*
Tolerant mutants					
1	Δ*recF*	DNA replication and repair protein RecF	DNA repair	+++	
2	Δ*metR*	HTH‐type transcriptional regulator MetR	Methionine synthesis	+	*
3	Δ*metE*	Homocysteine methyltransferase	Methionine synthesis	+	*
4	Δ*leuB*	3‐isopropylmalate dehydrogenase	l‐leucine biosynthesis	++	
5	Δ*leuL*	Leu operon leader peptide	l‐leucine biosynthesis	+	*
6	Δ*nadC*	Nicotinate‐nucleotide pyrophosphorylase	NAD^+^ synthesis	++	*
7	Δ*nadB*	l‐aspartate oxidase	NAD^+^ synthesis	++	*
8	Δ*glnE*	Bifunctional glutamine synthetase adenylyltransferase enzyme	Glutamine synthetase activity	+	
9	Δ*gshA*	Glutamate‐‐cysteine ligase	Glutathione biosynthesis	++	
10	Δ*ygfJ*	Molybdenum cofactor cytidylyltransferase	Molybdopterin cytosine dinucleotide biosynthesis	++	*
11	Δ*sspA*	Stringent starvation protein A	Stress response	++	

^a^ Tolerance levels were determined based on the relative cell survival ratio shown in Figure 1E,F. “−”: >0.01%; “−−”: <0.01%; “−−−”: <0.005%; “+”: ≥0.5%; “++”: ≥1%; and “+++”: ≥10%.

^b^ An asterisk (*) indicates that the corresponding gene has not previously been reported to be involved in antibiotic persistence.

Functional annotation analysis revealed that ciprofloxacin‐sensitive mutants were primarily enriched in DNA mismatch repair (e.g., *uvrD*) and homologous recombination repair pathways (e.g., *ruvA*, *ruvC*, *recG*, and *recN*). These findings are consistent with previous reports implicating DNA repair genes, e.g., *ruvA* and *ruvC*, in quinolone antibiotic tolerance^50^, ^51^. In addition, *holC* mutants have been reported to show increased sensitivity to DNA‐damaging agents^52^ and Δ*holC* was identified here as a ciprofloxacin‐sensitive mutant. Notably, *rnr* and *nfo* were also identified as DNA repair‐related genes, a conclusion further supported by protein–protein interaction analysis revealing close functional associations among DNA repair genes (Figure 1G). Moreover, multiple genes were found to be interconnected within energy metabolism‐related pathways (red dashed frame in Figure 1G).

Among the 11 tolerant mutants, minimum inhibitory concentrations (MICs) of ciprofloxacin were observed to be 0.03125 μg/ml, comparable to that of the wild‐type strain BW25113 (Figure S5A). Meanwhile, time‐dependent killing curves further demonstrated enhanced tolerance of these mutants relative to wild‐type cells (Figures 1H and S5B). Both characteristics are hallmarks of persister cells^20^. In contrast, five representative strains (Δ*fis*, Δ*nfo*, Δ*recN*, Δ*ruvC*, and Δ*rnr*) selected from the 37 sensitive mutants showed reduced MICs relative to BW25113 (Figure S5C). As MIC values were measured under 24‐h antibiotic exposure conditions, the reduced MICs observed in sensitive mutants reflect impaired antibiotic tolerance resulting from the corresponding gene deletions.

Functional annotation further indicated that tolerant mutants were distributed across several metabolic pathways (Table 1), including leucine biosynthesis (Δ*leuB* and Δ*leuL*) and methionine biosynthesis (Δ*metR* and Δ*metE*). Notably, two nicotinamide adenine dinucleotide (NAD⁺) biosynthesis mutants, Δ*nadB* and Δ*nadC*, were identified, a finding that contrasts with a previous transposon library screen reporting increased gentamicin sensitivity in *E. coli* Δ*nadB* and Δ*nadC* mutants^40^. Additionally, representative mutants were cultured under exponential‐phase conditions to generate type II persisters^20^ for antibiotic tolerance assays. Under these conditions, Δ*nadB*, Δ*nadC*, Δ*leuB*, and Δ*metR* no longer showed increased ciprofloxacin tolerance relative to wild‐type cells (Figure S6A), whereas two of the three sensitive mutants examined, Δ*ruvC* and Δ*rnr*, remained highly sensitive to ciprofloxacin (Figure S6B).

In summary, this genome‐wide screen revealed multifaceted biological pathways involved in the ciprofloxacin tolerance of *E. coli* persisters during resuscitation, identified several genes not previously associated with persistence, and highlighted candidates warranting further investigation.

### Ciprofloxacin‐sensitive mutants Δ*ruvC* and Δ*rnr* are not sensitive to ampicillin and gentamicin

Based on the enrichment of DNA repair‐related genes among ciprofloxacin‐sensitive mutants, Δ*ruvC* and Δ*rnr* were selected for further characterization. The Δ*ruvC* mutant was selected because of its established role in conferring low quinolone tolerance^50^, ^51^, an effect attributed to the loss of its essential endodeoxyribonuclease activity required for Holliday junction resolution^53^. In contrast, Δ*rnr* represents a previously unexplored candidate; its protein product has documented ribonuclease and helicase activities^54^ but has not been previously implicated in antibiotic tolerance.

**Figure 2:**
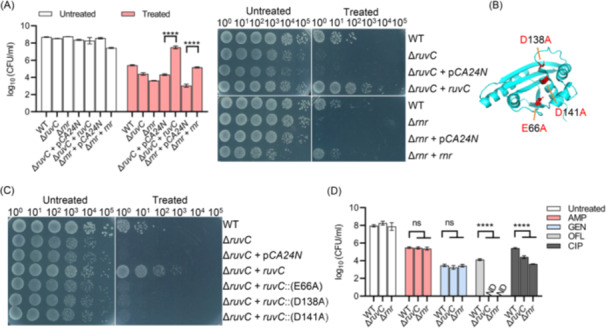
Characterization of ciprofloxacin‐specific sensitive mutants Δ*ruvC* and Δ*rnr*. (A) Survival of Δ*ruvC*, Δ*rnr*, and their complemented strains following 3 h of treatment with 2.5 μg/ml ciprofloxacin during resuscitation. Left panel: quantified survival results; right panel: representative images of cell survival on LB agar plates. The growth defect observed in the Δ*rnr*+*rnr* strain is likely attributable to side effects of complementary Rnr expression at non‐physiological levels or other unknown factors. (B) Three‐dimensional structure of RuvC is derived from the Protein Data Bank (PDB), and the PDB ID is 1HJR. Three amino acid residues previously reported to be essential for the endodeoxyribonuclease activity of RuvC⁵⁵ are highlighted in red. (C) Survival of Δ*ruvC* cells following complementary expression of the indicated RuvC mutant proteins and subsequent 3 h treatment with 2.5 μg/ml ciprofloxacin during resuscitation. (D) Survival of Δ*ruvC* and Δ*rnr* cells following 3 h treatment with 200 μg/ml ampicillin (AMP), 7.5 μg/ml gentamicin (GEN), 2.5 μg/ml ciprofloxacin (CIP), or 2.5 μg/ml ofloxacin (OFL). ND indicates that no CFUs were detectable on LB agar plates. All experiments were performed in triplicate. Error bars represent standard errors. Results of Analysis of Variance with Tukey's HSD post hoc tests are shown; ****p < 0.0001; ns, not significant.

Initially, it was demonstrated that complementary expression of Rnr and RuvC (Figures S7A,B) fully restored the ciprofloxacin tolerance of Δ*rnr* and Δ*ruvC* mutants, respectively, to similar or even higher levers than BW25113 cells (WT) (Figure 2A). Next, three point‐mutant variants of RuvC (E66A, D138A, and D141A), all of which abolish enzymatic activity^55^, were constructed. As expected, complementary expression of these inactive RuvC variants failed to restore ciprofloxacin tolerance in the Δ*ruvC* mutant (Figure 2B,C), confirming that the reduced tolerance of Δ*ruvC* might be directly attributable to impaired DNA repair function. Furthermore, the sensitivity of these mutants to other classes of bactericidal antibiotics was assessed. Cell survival assays revealed that both Δ*ruvC* and Δ*rnr* mutants were more sensitive to ofloxacin, another quinolone antibiotic, than BW25113. In contrast, neither mutant showed increased sensitivity to the β‐lactam antibiotic ampicillin or the aminoglycoside antibiotic gentamicin (Figure 2D). Consistently, both Δ*ruvC* and Δ*rnr* mutants showed lower MICs for ciprofloxacin (Figure S5C), but unchanged MICs for ampicillin and gentamicin (Figure S7D), relative to the wild type, accounting for the specific hypersensitivity to ciprofloxacin.

These observations suggest that alterations in persister tolerance caused by deletion of DNA repair genes are largely specific to quinolone antibiotics, consistent with DNA topoisomerase and gyrase being primary targets of quinolones^56^. Notably, the Δ*recF* mutant, which lacks the DNA replication and repair protein RecF, was conversely more tolerant to ciprofloxacin (Figure S7E), in agreement with previous finding^50^. This mutant also failed to show tolerance to ampicillin or gentamicin (Figure S7E). In addition, ciprofloxacin sensitivity in Δ*recN* and Δ*nfo* mutants was reversed upon complementary expression of RecN and Nfo, respectively (Figure S7F and lanes 11–15 in Figure S7A). By contrast, complementary expression of HolC and Fis in their respective mutants had minimal effects on ciprofloxacin tolerance, and expression of RuvA further increased ciprofloxacin sensitivity (Figure S7A,F), likely due to improper protein folding and loss of functional activity. Consequently, the *fis* and *holC* mutants were not further investigated in this study.

### Ciprofloxacin‐tolerant mutants deficient in methionine, leucine, and NAD⁺ biosynthesis are tolerant to ampicillin and gentamicin

Genes involved in amino acid biosynthesis have been widely implicated in bacterial persister formation^15^, ^16^, ^40^. Consistent with these reports, four amino acid biosynthesis‐related genes, that is, *metR*, *metE*, *leuB*, and *leuL*, were identified as contributors to increased antibiotic tolerance. MetR functions as a positive transcriptional regulator of *metE*, which encodes an enzyme essential for methionine biosynthesis^57^. LeuB is a structural component of the leucine operon and encodes isopropylmalate dehydrogenase, a key enzyme in leucine biosynthesis, whereas LeuL is a leader peptide that regulates leucine biosynthetic gene expression^58^, ^59^.

**Figure 3:**
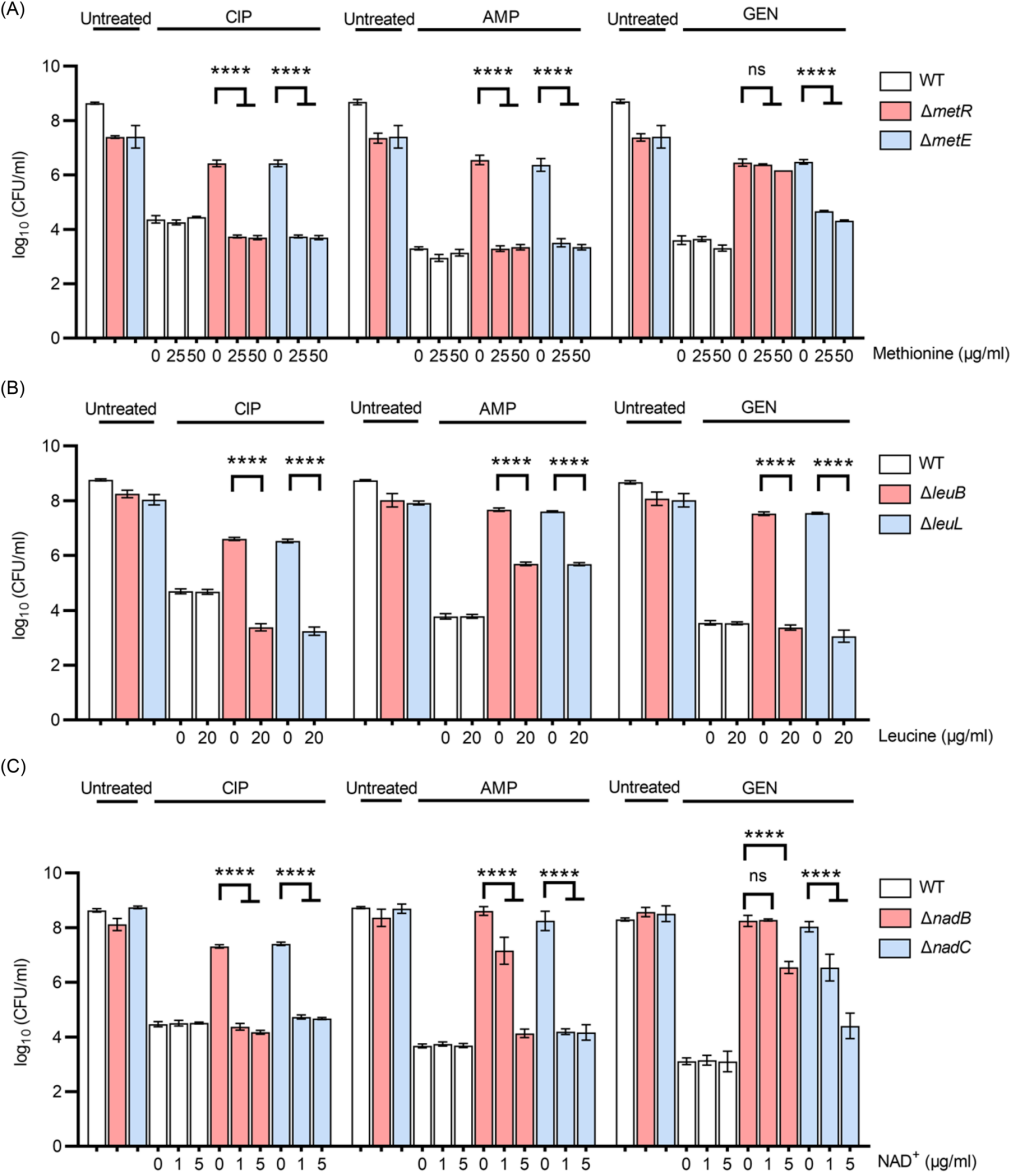
Characterization of amino acid and NAD⁺ biosynthesis‐deficient mutants. (A–C) Survival of methionine‐related mutants (Δ*metR* and Δ*metE*) (A), leucine‐related mutants (Δ*leuB* and Δ*leuL*) (B), and NAD⁺ biosynthesis‐related mutants (Δ*nadB* and Δ*nadC*) (C) subjected to treatment with ciprofloxacin (CIP, 2.5 μg/ml, 5 h), ampicillin (AMP, 200 μg/ml, 6 h), or gentamicin (GEN, 7.5 μg/ml, 3 h), respectively. Following antibiotic treatment, cells were resuscitated in fresh medium supplemented with methionine (0, 20, or 50 μg/ml) (A), leucine (0 or 20 μg/ml) (B), or NAD⁺ (0, 1, or 5 μg/ml) (C) at the indicated concentrations. All experiments were performed in triplicate. Error bars represent standard errors. Results of Analysis of Variance with Tukey's HSD post hoc tests are shown; ****p < 0.0001; ns, not significant.

It was first confirmed that complementary expression of MetR and LeuB significantly reduced ciprofloxacin tolerance in the corresponding mutants (Figure S8A,B). Second, supplementation of trace amounts of methionine in the resuscitation medium markedly decreased ciprofloxacin tolerance in Δ*metR* and Δ*metE* mutants (Figure 3A), and a similar effect was observed upon leucine supplementation in Δ*leuB* and Δ*leuL* mutants (Figure 3B). Notably, supplementation of methionine during growth rather than resuscitation did not reduce ciprofloxacin tolerance in Δ*metR* unless high concentrations were applied (Figure S8C), indicating that elevated tolerance results from methionine deficiency during persister resuscitation rather than during persister formation. Third, tolerance to other bactericidal antibiotics was assessed, revealing that all four mutants were more tolerant to ampicillin and gentamicin than BW25113 (Figure 3A,B). Supplementation of methionine or leucine in the resuscitation medium similarly reduced tolerance to ampicillin and gentamicin (with the exception of Δ*metR*), mirroring the effects observed with ciprofloxacin. In contrast, these supplements had negligible effects on antibiotic tolerance in wild‐type cells. In the case of Δ*metR*, methionine supplementation reduced gentamicin tolerance when antibiotic exposure was extended from 3 to 5 h (Figures 3A and S8D), suggesting additional regulatory functions of MetR beyond methionine biosynthesis. Collectively, these data indicate that disruption of methionine and leucine biosynthesis is critical for broad‐spectrum antibiotic tolerance in *E. coli* persisters during resuscitation, in contrast to DNA repair genes, which appear to confer quinolone‐specific tolerance.

In addition to amino acid biosynthesis, two NAD⁺ biosynthesis mutants, Δ*nadB* and Δ*nadC*, were identified as showing increased ciprofloxacin tolerance. Complementary expression of NadB and NadC significantly reduced ciprofloxacin tolerance in these mutants (Figure S8E,F). Furthermore, supplementation of 5 μg/ml NAD⁺ in the resuscitation medium markedly decreased ciprofloxacin tolerance in both mutants (Figure 3C). Similar to amino acid biosynthesis mutants, Δ*nadB* and Δ*nadC* also showed increased tolerance to ampicillin and gentamicin relative to BW25113 (Figure 3A–C). This tolerance was reduced upon NAD⁺ supplementation (Figure 3C), although only partial restoration of gentamicin sensitivity was observed, suggesting that NadB and NadC may influence gentamicin activity through mechanisms beyond NAD⁺ biosynthesis. Consistently, Δ*metR* and Δ*nadB* mutants also displayed tolerance to the clinically relevant antibiotic meropenem (Figure S8G). Together, these results establish that functional NAD⁺ biosynthesis is critical for antibiotic tolerance in *E. coli* persisters during resuscitation, consistent with the essential role of NAD⁺ as a cofactor for numerous enzymatic reactions.

### Ciprofloxacin‐sensitive mutants Δ*atpC* and Δ*atpF* possess elevated intracellular ATP levels

**Figure 4:**
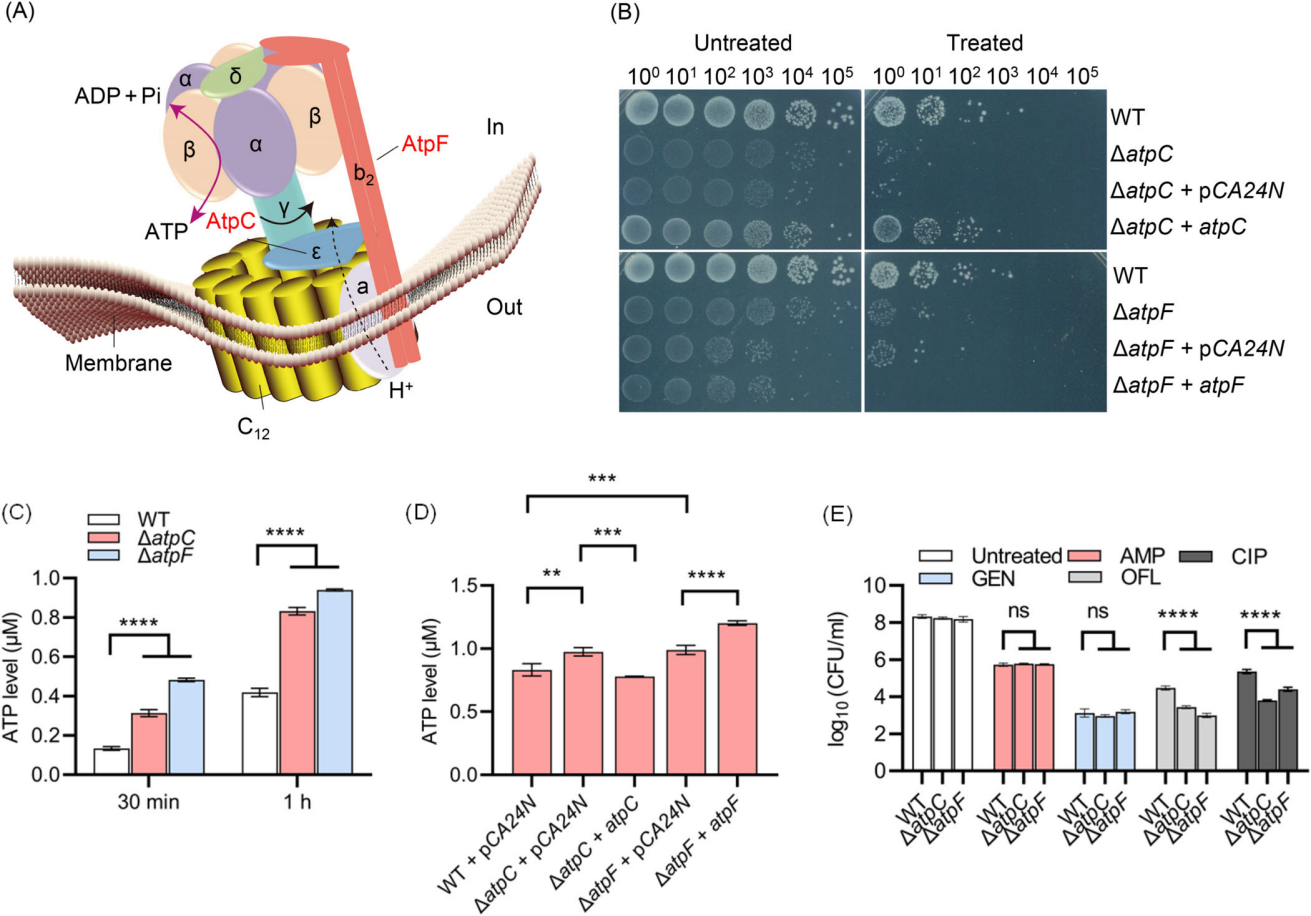
ATP synthase‐deficient mutants Δ*atpC* and Δ*atpF* show reduced ciprofloxacin tolerance and elevated intracellular ATP levels. (A) Schematic representation of the ATP synthase complex was drawn based on the crystal structure of ATP synthase proposed by J. Walke et al. *atpC* and *atpF* encode the ε and b_2_ subunits, respectively, which inhibit ATP hydrolysis and are responsible for linking the *F*₀ and *F*₁ subcomplexes. (B) Survival of Δ*atpC*, Δ*atpF*, and their complemented strains following 3 h of treatment with 2.5 μg/ml ciprofloxacin during resuscitation. (C) Intracellular ATP levels of wild‐type, Δ*atpC*, and Δ*atpF* cells following 0.5 or 1 h of resuscitation. Cell densities were normalized prior to resuscitation. (D) Intracellular ATP levels of Δ*atpC*, Δ*atpF*, and their complemented strains following 1 h of resuscitation. (E) Survival of Δ*atpC* and Δ*atpF* cells following 3 h of treatment with 200 μg/ml AMP, 7.5 μg/ml GEN, 2.5 μg/ml CIP, or 2.5 μg/ml OFL. All experiments were performed in triplicate. Error bars represent standard errors. Results of Analysis of Variance with Tukey's HSD post hoc tests are shown; **p < 0.01; ***p < 0.001; ****p < 0.0001; and ns, not significant.

Among the ciprofloxacin‐sensitive mutants identified, Δ*atpC* and Δ*atpF*, which lack the ε and b_2_ subunits of ATP synthase, respectively, were selected for further investigation for several reasons. Importantly, ATP has been widely recognized as a determinant of persister formation and antibiotic tolerance^44^, ^60^, ^61^, ^62^, although its effects have predominantly been examined through chemical perturbation. In addition, the ε and b_2_ subunits of ATP synthase play regulatory and structural roles, respectively, rather than acting as catalytic subunits for ATP synthesis or hydrolysis^63^, ^64^ (Figure 4A). Notably, Δ*atpF* was previously reported^35^ to be enriched following repeated ampicillin treatment in an *E. coli* transposon‐mutagenized library, a finding that contrasts with the ciprofloxacin sensitivity observed here. Furthermore, deletion of *atpA*, encoding the α regulatory subunit, has also been reported to reduce antibiotic tolerance^65^.

Complementary expression of AtpC restored ciprofloxacin tolerance in Δ*atpC* to levels comparable to BW25113 (upper panels in Figures 4B and S9A). In contrast, complementary expression of AtpF in Δ*atpF* further decreased ciprofloxacin tolerance rather than restoring it (lower panels in Figures 4B and S9B). Given the established role of ATP in antibiotic tolerance^60^, ^66^, it was hypothesized that this unexpected phenotype might be associated with altered intracellular ATP levels. Indeed, ATP measurements revealed that intracellular ATP levels in both Δ*atpC* and Δ*atpF* mutants were higher than those in BW25113 (Figure 4C), and that ATP levels in the Δ*atpC*+*atpC* strain were reduced relative to Δ*atpC*, consistent with their respective ciprofloxacin tolerance phenotypes (Figure 4B). Notably, ATP levels in both BW25113 and the mutants were significantly higher at 1 h of resuscitation than at 0.5 h.

Unlike the Δ*atpC*+*atpC* strain, ATP levels in the Δ*atpF*+*atpF* strain were further elevated relative to Δ*atpF* (Figure 4D), providing a mechanistic explanation for the increased ciprofloxacin sensitivity of Δ*atpF*+*atpF* (Figure 4B), assuming that elevated ATP levels correspond to reduced antibiotic tolerance^60^, ^62^, ^66^. It is proposed that ectopic AtpF expression fine‐tunes the ATP synthase complex, potentially shifting its conformational equilibrium toward enhanced ATP synthesis^67^, thereby abnormally increasing intracellular ATP levels. Additionally, both Δ*atpC* and Δ*atpF* mutants were found to be sensitive to ofloxacin and ciprofloxacin, but were resistant to both ampicillin and gentamicin (Figure 4E). Consistently, Δ*atpC* and Δ*ruvC* mutants were not sensitive to meropenem (Figure S9C). Importantly, mutants defective in the tricarboxylic acid cycle (Δ*sucC* and Δ*sucD*) or the respiratory chain (Δ*ndh*, Δ*nuoB*, and Δ*nuoG*) did not show increased ciprofloxacin tolerance (Figure S10A) or altered ATP levels (Figure S10B). Collectively, these findings strongly support the conclusion that intracellular ATP level is a major determinant of quinolone tolerance in ATP synthase‐modified *E. coli* strains.

### A negative correlation between ATP level and ciprofloxacin tolerance for *E. coli* persisters

**Figure 5:**
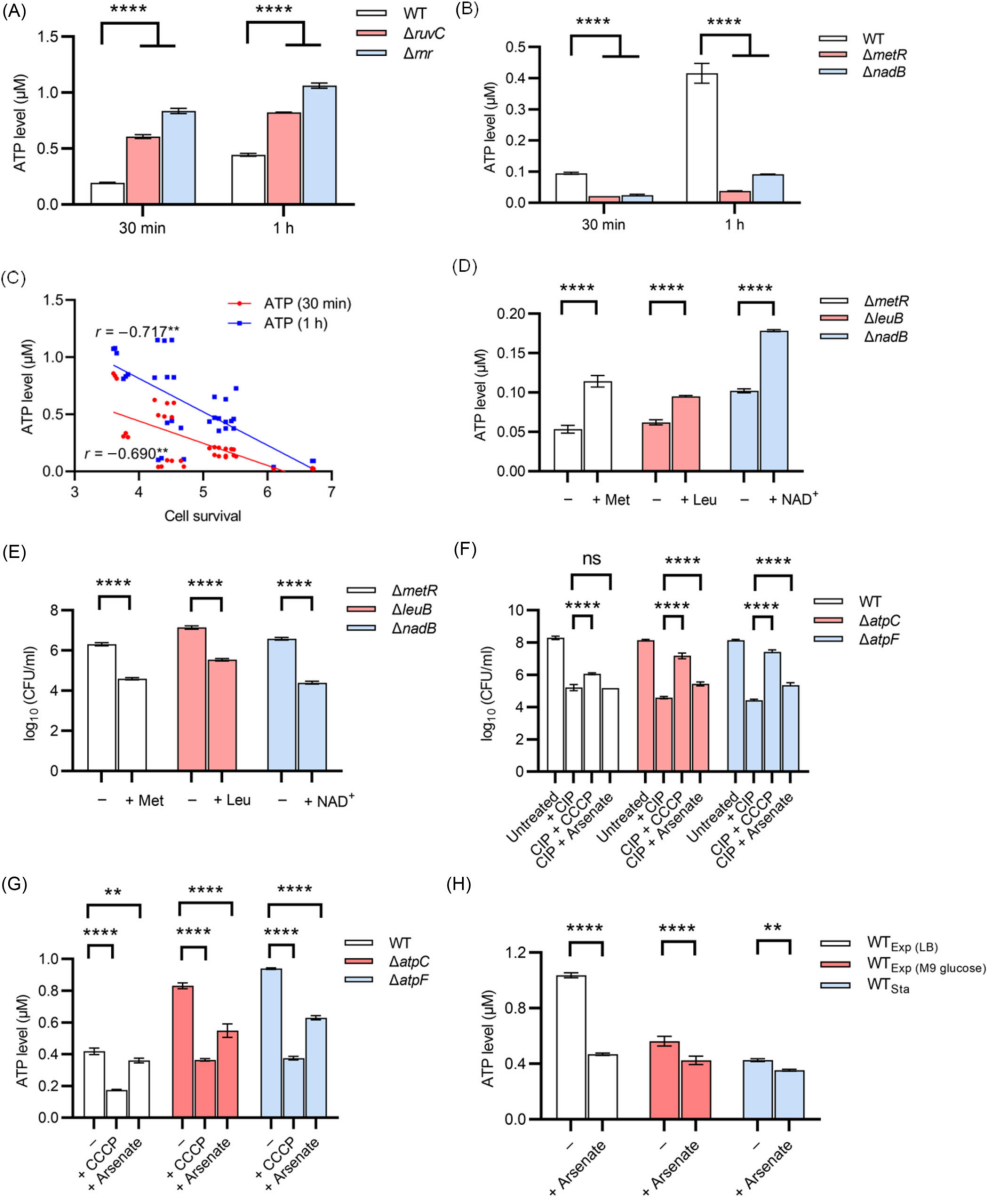
Negative correlation between intracellular ATP levels and ciprofloxacin tolerance in genetically distinct *Escherichia coli* persisters during resuscitation. (A, B) Intracellular ATP levels of Δ*ruvC* and Δ*rnr* cells (A) and Δ*metR* and Δ*nadB* cells (B) following 0.5 or 1 h of resuscitation. Cell densities of wild‐type cells were adjusted to match those of the corresponding mutant cells prior to resuscitation. (C) Negative correlation between intracellular ATP levels and cell survival among genetically distinct *E. coli* strains, including wild type, Δ*ruvC*, Δ*rnr*, Δ*metR*, Δ*nadB*, Δ*atpC*, and Δ*atpF*. Data were derived from panels A and B and from Figure 4C. Pearson correlation coefficients were calculated as −0.717 and −0.690 (**p < 0.01). (D, E) Intracellular ATP level (D) and cell survival (E) of Δ*metR*, Δ*leuB*, and Δ*nadB* cells following 2 h of resuscitation in the presence of 25 μg/ml methionine (Met), 20 μg/ml leucine (Leu), or 5 μg/ml β‐nicotinamide adenine dinucleotide (NAD⁺), respectively. Ciprofloxacin (2.5 μg/ml) was added in panel E for antibiotic treatment. (F) Survival of wild‐type, Δ*atpC*, and Δ*atpF* cells following 3 h of treatment with 2.5 μg/ml ciprofloxacin in the presence of 10 mM arsenate or 20 μM carbonyl cyanide 3‐chlorophenyl hydrazone (CCCP). (G) Intracellular ATP levels of wild‐type, Δ*atpC*, and Δ*atpF* cells following 1 h of resuscitation in the presence of 10 mM arsenate or 20 μM CCCP. (H) Intracellular ATP levels of wild‐type *E. coli* cells in exponential and stationary phases following 1 h of treatment with 10 mM arsenate. Arsenate was directly added to exponential‐phase cultures and to stationary‐phase cultures following 20‐fold dilution into fresh M9 glucose. All experiments were performed in triplicate. Error bars represent standard errors. Results of Analysis of Variance with Tukey's HSD post hoc tests are shown; **p < 0.01; ****p < 0.0001; and ns, not significant.

The correlation between intracellular ATP levels and quinolone tolerance was next extended to other identified mutants. It was found that Δ*ruvC* and Δ*rnr*, as representative ciprofloxacin‐sensitive mutants, showed higher intracellular ATP levels than BW25113 during resuscitation for 0.5 or 1 h (Figure 5A). Conversely, Δ*metR* and Δ*nadB*, as representative ciprofloxacin‐tolerant mutants, showed lower intracellular ATP levels than BW25113 under the same conditions (Figure 5B). Again, ATP levels increased in each strain during resuscitation (Figure 5A,B). Quantitative analysis of ATP levels and cell survival in Δ*atpC*, Δ*atpF*, Δ*ruvC*, Δ*rnr*, Δ*metR*, and Δ*nadB* mutants revealed a negative correlation between these variables (Figure 5C), with correlation coefficients reaching −0.717 and −0.690 based on the 1 and 0.5 h datasets, respectively. In further support of this conclusion, ciprofloxacin tolerance recovery in Δ*metR*, Δ*leuB*, and Δ*nadB* mutants was examined following supplementation of methionine, leucine, and NAD⁺ in the resuscitation medium, respectively, as described above (Figure 3). Under these conditions, ATP levels in these mutants were significantly increased upon addition of the corresponding metabolite (Figure 5D), and ciprofloxacin tolerance was concomitantly decreased (Figure 5E).

A chemical inhibition strategy was also applied, in which intracellular ATP levels were altered by treatment with arsenate^60^, ^68^, and carbonyl cyanide 3‐chlorophenyl hydrazone (CCCP)^62^, ^67^, to further evaluate the relationship between ATP levels and antibiotic tolerance. Cell survival assays showed that the ciprofloxacin‐sensitive mutants Δ*atpC* and Δ*atpF* became more tolerant to ciprofloxacin upon treatment with arsenate or CCCP during resuscitation, with CCCP producing a stronger effect than arsenate (Figure 5F). Consistent with these phenotypic changes, ATP levels in these mutants were decreased to varying degrees, with ATP levels in CCCP‐treated cells lower than those in arsenate‐treated cells for each mutant (Figure 5G).

Notably, although ATP levels in wild‐type BW25113 were significantly decreased by arsenate treatment (Figure 5G), ciprofloxacin tolerance was not significantly enhanced (Figure 5F). This result differs from a previous report by the Lewis group⁶⁰, likely because stationary‐phase persisters were used in the present model, whereas exponential‐phase cells were used in the cited study. To address this discrepancy, it was demonstrated that arsenate markedly decreased ATP levels and increased ciprofloxacin tolerance in exponential‐phase BW25113 cells cultured in LB medium (Figures 5H and S11A), thereby confirming the earlier findings^60^. When cultures were shifted to M9 glucose medium, arsenate‐induced ATP reduction and increases in ciprofloxacin tolerance were also detected in exponential‐phase BW25113 cells, although to a lesser extent. By contrast, the absolute ATP level observed after 1 h resuscitation from the stationary phase in the present model was lower than that in exponential‐phase cultures and was only modestly, albeit significantly, reduced by arsenate (Figure 5H). It is proposed that this limited reduction was insufficient to substantially enhance ciprofloxacin tolerance during resuscitation. Consistent with the differential efficacy of these inhibitors, CCCP, but not arsenate, significantly delayed the growth of wild‐type cells, whereas both compounds markedly impaired the growth of Δ*atpC* and Δ*atpF* mutants (Figure S11B).

### Switch of M9 glucose to LB medium decreases ciprofloxacin tolerance and increases ATP levels in persisters

**Figure 6:**
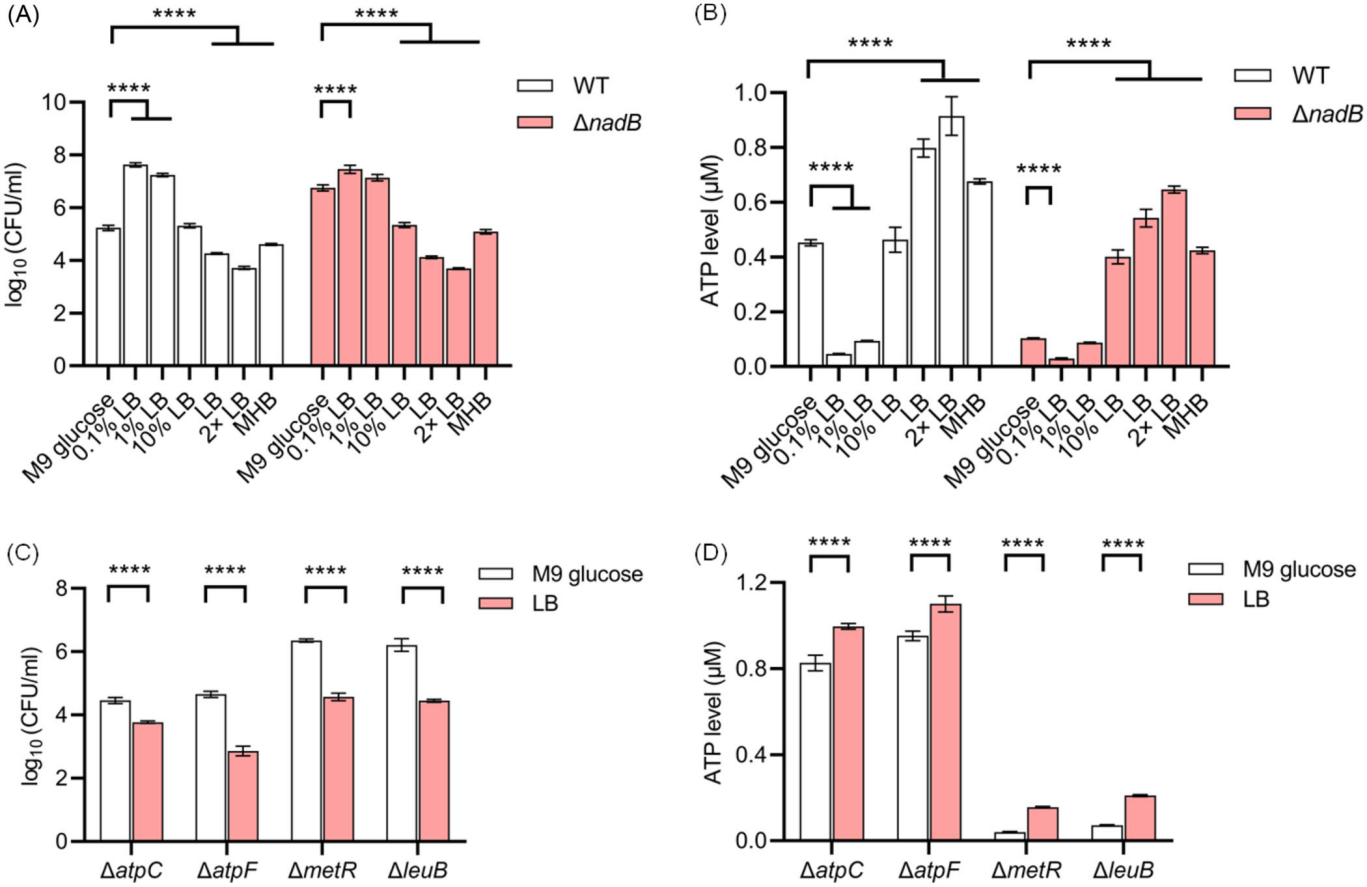
Switching from M9 glucose medium to LB medium during resuscitation decreases ciprofloxacin tolerance and increases intracellular ATP levels in *E. coli*. (A) Survival of wild‐type and Δ*nadB* cells following 3 h of treatment with 2.5 μg/ml ciprofloxacin during resuscitation in the indicated media (M9 glucose, 0.1% LB, 1% LB, 10% LB, LB, 2× LB, and MHB). (B) Intracellular ATP levels of wild‐type and Δ*nadB* cells following 1 h of resuscitation in the indicated media. (C) Survival of ciprofloxacin‐sensitive mutants (Δ*atpC* and Δ*atpF*) and ciprofloxacin‐tolerant mutants (Δ*metR* and Δ*leuB*) following 3 h of treatment with 2.5 μg/ml ciprofloxacin during resuscitation in M9 glucose or LB medium. (D) Intracellular ATP levels of ciprofloxacin‐sensitive mutants (Δ*atpC* and Δ*atpF*) and ciprofloxacin‐tolerant mutants (Δ*metR* and Δ*leuB*) following 1 h of resuscitation in M9 glucose or LB medium. All experiments were performed in triplicate. Error bars represent standard errors. Results of Analysis of Variance with Tukey's HSD post hoc tests are shown; ****p < 0.0001.

A conventional strategy to enhance ciprofloxacin efficacy against *E. coli* persisters was next investigated. Motivated by earlier reports indicating that ATP levels in *E. coli* cells are positively correlated with LB concentration during culturing^69^, ^70^, it was observed that ciprofloxacin tolerance of BW25113 decreased progressively upon resuscitation in increasing concentrations of LB medium (Figure 6A), while intracellular ATP levels increased concomitantly (Figure 6B). Similar results were obtained for the ciprofloxacin‐tolerant mutant Δ*nadB* (Figure 6A,B). Consistently, switching from M9 glucose medium to LB medium rendered ciprofloxacin‐tolerant mutants Δ*metR* and Δ*leuB* more sensitive to ciprofloxacin (Figure 6C) and increased their ATP levels (Figure 6D). Notably, LB‐induced potentiation of ciprofloxacin efficacy and elevation of ATP levels were also observed in ciprofloxacin‐sensitive mutants Δ*atpC* and Δ*atpF* (Figure 6C,D). These findings suggest that manipulation of nutritional abundance may represent an efficient strategy to modulate *E. coli* persister antibiotic tolerance through alteration of intracellular ATP levels. Furthermore, supplementation with casamino acids, rather than LB medium, significantly restored ciprofloxacin sensitivity in Δ*metR*, although minimal effects were observed in Δ*nadB* (Figure S12A) and other ciprofloxacin‐sensitive mutants (Figure S12B), thereby indicating the potential therapeutic value of casamino acids in reducing antibiotic persistence.

### Sensitive and tolerant mutants show higher and lower levels of antibiotic uptake and reactive oxyen species (ROS), respectively

**Figure 7:**
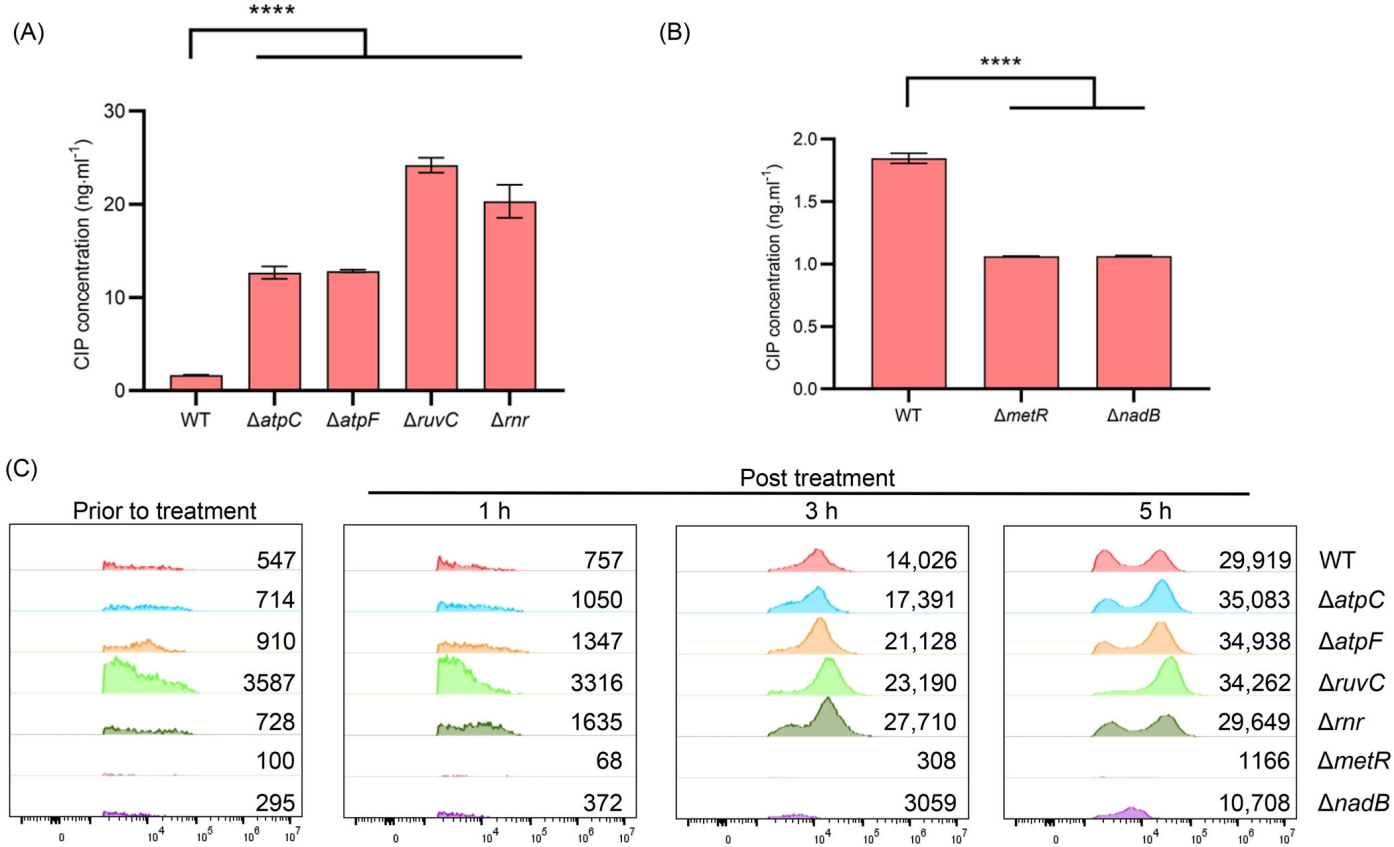
Correlation of ciprofloxacin persistence with antibiotic uptake and reactive oxygen species (ROS) levels. (A) Intracellular ciprofloxacin concentrations in wild‐type cells and ciprofloxacin‐sensitive mutants (Δ*atpC*, Δ*atpF*, Δ*ruvC*, and Δ*rnr*) following 3 h of treatment with 2.5 μg/ml ciprofloxacin during resuscitation. (B) Intracellular ciprofloxacin concentrations in wild‐type cells and ciprofloxacin‐tolerant mutants (Δ*metR* and Δ*nadB*) following 5 h of treatment with 2.5 μg/ml ciprofloxacin during resuscitation. (C) Flow cytometric analysis of ROS levels in the indicated mutant strains (Δ*atpC*, Δ*atpF*, Δ*ruvC*, Δ*rnr*, Δ*metR*, and Δ*nadB*) and the wild‐type strain prior to ciprofloxacin treatment (left panel) and following treatment (middle and right panels), as detected using dihydroethidium (DHE). The numbers of fluorescence‐positive cells among a total of 50,000 analyzed cells are indicated. All experiments were performed in triplicate. Error bars represent standard errors. Results of Analysis of Variance with Tukey's HSD post hoc tests are shown; ****p < 0.0001.

To elucidate the mechanism by which ATP influences ciprofloxacin persistence, intracellular ciprofloxacin accumulation was first compared between wild‐type and mutant strains. As shown in Figure 7A, intracellular ciprofloxacin levels in ciprofloxacin‐sensitive mutants (Δ*atpC*, Δ*atpF*, Δ*ruvC*, and Δ*rnr*) were approximately 10‐fold higher than those in wild‐type cells following 3 h of antibiotic treatment. Conversely, ciprofloxacin levels in ciprofloxacin‐tolerant mutants (Δ*metR* and Δ*nadB*) were approximately half of those in the wild‐type strain (Figure 7B). These results indicate that ATP‐associated differences in ciprofloxacin persistence are associated with ciprofloxacin uptake.

ROS levels were also compared, as ROS has been proposed as a shared mechanism contributing to the lethal actions of bactericidal antibiotics^71^, ^72^, ^73^. Flow cytometric analyses revealed that Δ*atpC*, Δ*atpF*, Δ*ruvC*, and Δ*rnr* showed higher ROS levels than the wild‐type strain following 1 h and 3 h of antibiotic treatment, although ROS levels among these mutants were broadly comparable after 5 h of treatment (Figure 7C). In contrast, Δ*metR* and Δ*nadB*, as ciprofloxacin‐tolerant mutants, maintained substantially lower ROS levels than wild‐type cells. Notably, basal ROS levels (prior to antibiotic exposure) were higher in Δ*ruvC* cells than in the other strains (Figure 7C), whereas ROS levels in Δ*metR* remained extremely low regardless of antibiotic treatment (Figure 7C). Collectively, these findings indicate that ATP‐modulated ciprofloxacin persistence is also associated with ROS accumulation.

## DISCUSSION

### Discovery of numerous genes involved in the ciprofloxacin tolerance of *E. coli* persisters

Multiple biological pathways have been reported to participate in the formation and survival of bacterial persisters, based on evidence from classical genetic screens, candidate‐based approaches, and physiological studies^16^. These pathways include, but are not limited to, toxin–antitoxin modules, energy metabolism, protein degradation systems and trans‐translation, purine and amino acid metabolism, DNA repair and the SOS response, quorum sensing, the stringent response and ppGpp signaling, efflux pump systems, epigenetic modifications, general stress responses and global regulators, as well as pathways involved in biofilm formation^16^. In the present study, 48 genes associated with ciprofloxacin tolerance in *E. coli* were identified, most of which fall within these established functional categories. Notably, 25 of these genes have not been previously associated with bacterial persistence (Table 1), underscoring the effectiveness of the screening strategy used, which clearly separates persister formation from the resuscitation and antibiotic treatment phases.

It should be noted that removal of the kanamycin resistance cassette in Δ*atpC*, Δ*atpF*, Δ*metE*, Δ*nadC*, Δ*leuB*, and Δ*leuL* mutants did not alter their ciprofloxacin tolerance phenotypes (Figure S13A,B), thereby excluding polar effects of the kanamycin cassette in the Keio collection. In addition, single‐gene deletion mutants were independently constructed in the BW25113 background using CRISPR‐based methods as previously described^74^, ^75^. These independently generated mutants, such as Δ*atpC* and Δ*ruvC* as representative sensitive mutants and Δ*metR* and Δ*nadB* as representative tolerant mutants, showed ciprofloxacin tolerance phenotypes consistent with those observed in the corresponding Keio mutants (Figure S13C–E). Nevertheless, certain well‐characterized ciprofloxacin‐sensitive genes, for example, *recA* (Figure S13F), were not identified in the initial screen (Figure S2A), indicating that the screening approach used has limitations in comprehensively capturing all genes involved in antibiotic persistence.

Genes associated with DNA repair and the SOS response have been widely reported to contribute to persistence of *E. coli* cells, particularly in response to quinolone antibiotics^15^, ^76^. Accordingly, it is not surprising that DNA repair‐related genes, for example, *uvrD* ^77^, ^78^, *ruvA*, *ruvC*, *recG,* ^79^ and *recN* ^80^, were identified in this study, consistent with previous reports. Notably, several DNA repair‐associated genes, including *rnr*, *nfo*, and *holC*, were identified here for the first time in the context of persister antibiotic tolerance, further highlighting the importance of DNA repair pathways in quinolone tolerance. Rnr is a ribonuclease involved in rRNA maturation or degradation and mRNA turnover^81^, and also possesses helicase activity^54^. The precise mechanisms by which Rnr influences ciprofloxacin tolerance in *E. coli* therefore warrant further investigation. Nfo functions in the recognition and cleavage of apurinic/apyrimidinic sites in DNA and thus participates in base excision repair^82^. HolC is a subunit of the DNA polymerase III complex and plays roles in DNA replication and repair^83^.

Amino acid biosynthesis has been well established to influence persister formation and antibiotic tolerance through mechanisms, for example, translational arrest, modulation of proton motive force, and activation of the ppGpp‐mediated stringent response^15^. In the present study, both previously reported and newly identified amino acid biosynthesis‐related genes, including *metR*, *metE*, and *leuL*, were associated with increased ciprofloxacin tolerance (Table 1). It is proposed that the elevated tolerance observed in these biosynthetic mutants operates through mechanisms analogous to those previously described^15^.

NAD⁺, a central redox cofactor for numerous metabolic enzymes, is essential for cellular metabolism and physiological homeostasis. In this study, the NAD⁺ biosynthesis genes *nadB* and *nadC* were identified as persister‐related genes, and exogenous supplementation with NAD⁺ was shown to restore ciprofloxacin sensitivity in the corresponding mutants. In addition, several genes belonging to other biological processes were identified, including outer membrane biogenesis (*yfgL*, also known as *bamB*), enterobacterial common antigen biosynthesis (*rffC*), cell division (*minC*), lysozyme inhibition (*ivy*), and phosphate transport (*phnH*). Elucidation of the mechanisms by which these diverse genes modulate antibiotic tolerance during persister resuscitation represents an important avenue for future research and is currently being actively pursued.

### A universal role of ATP in ciprofloxacin persistence

Whether a general mechanism underlies persister formation and antibiotic tolerance, and whether a universal molecular marker exists to indicate the persister state of bacterial cells remain fundamental questions in the field. Cell growth has generally been considered a determinant of persister formation and antibiotic persistence^84^. However, it has been reported that “dormancy is not necessary or sufficient for bacterial persistence”^85^. In support of this conclusion, five of the 11 ciprofloxacin‐tolerant mutants identified in this study showed growth rates comparable to those of the wild‐type strain (Figure S14A), whereas the remaining six mutants displayed severe growth defects in M9 glucose medium (Figure S14B). Supplementation of amino acids or NAD⁺ in the growth medium of these non‐growing mutants only partially restored growth (Figure S14B), yet fully restored ciprofloxacin sensitivity (Figure 3). Moreover, systematic analysis of gene deletion mutants in the histidine biosynthesis pathway (Δ*hisA*, Δ*hisB*, Δ*hisC*, Δ*hisD*, Δ*hisF*, Δ*hisG*, and Δ*hisI*) revealed varying degrees of growth impairment relative to the wild‐type strain, but no corresponding increase in ciprofloxacin tolerance (Figure S14C,D). Notably, these histidine biosynthesis mutants showed differential tolerance to ampicillin (Figure S14D). Collectively, these results indicate that growth rate alone is not an absolute determinant of persister formation, a conclusion that also extends to other proposed mechanisms such as toxin–antitoxin modules, ppGpp signaling, and the stringent response, which appear to be context‐ and species‐dependent.

A low intracellular ATP level has been proposed as a unifying mechanism for persister formation and antibiotic tolerance^66^, based on several key observations: (i) *Staphylococcus aureus* cells in the stationary phase show high antibiotic tolerance and ATP levels approximately tenfold lower than those of exponential‐phase cells, and (ii) arsenate treatment depletes ATP levels in exponential‐phase cells of *S. aureus*, *E. coli*, and *P. aeruginosa*, concomitantly increasing antibiotic tolerance^38^, ^60^, ^86^. This low‐energy hypothesis provides a simple and plausible explanation for the formation of diverse types of persisters, assuming that reduced ATP levels lead to decreased activity of antibiotic targets. In the present study, a strong negative correlation between intracellular ATP levels and ciprofloxacin tolerance was observed across genetically diverse *E. coli* mutants (Figure 5C), although the precise mechanisms by which individual gene deletions alter ATP levels remain unclear. This negative correlation was consistently observed following chemical inhibition with arsenate or CCCP (Figure 5F–H), metabolite supplementation (Figure 5D,E), and nutrient switching from M9 glucose to LB medium (Figure 6). Notably, specialized persisters induced by expression of TisB or HokB also conform to the low ATP–high antibiotic tolerance paradigm due to ATP leakage through membrane pores^87^. In addition, deletion of the global regulator *ihfA* was reported to result in reduced ciprofloxacin tolerance and elevated ATP levels, regardless of carbon source availability^88^. Collectively, these findings constitute some of the most comprehensive evidence to date supporting low intracellular ATP as a general mechanism unifying persister formation and survival across diverse experimental models.

**Figure 8:**
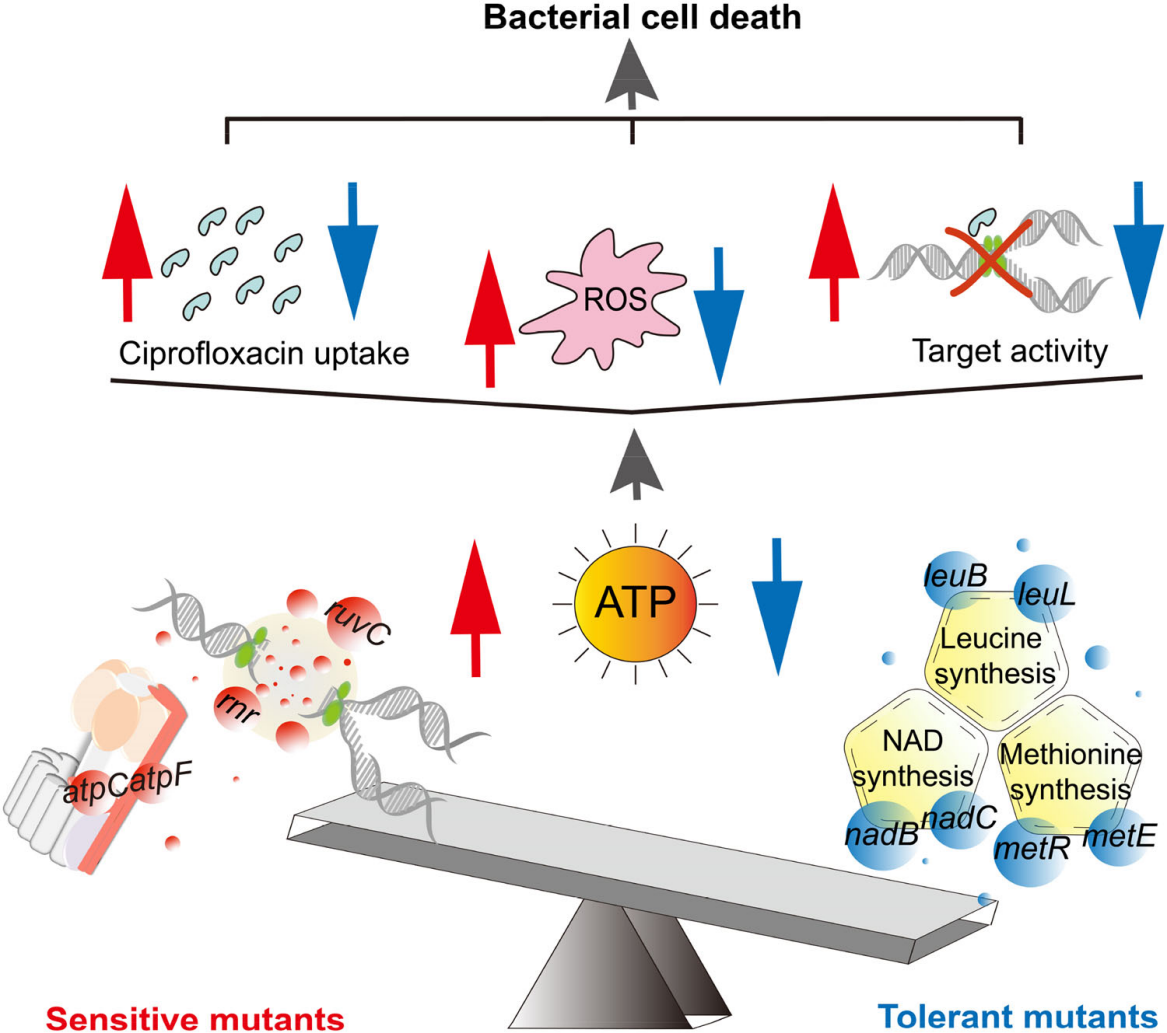
Schematic illustration of ATP‐regulated ciprofloxacin persistence in genetically distinct persisters. Ciprofloxacin‐sensitive mutants show elevated intracellular ATP levels, which in turn enhance ciprofloxacin uptake and promote increased ROS accumulation, thereby rendering the antibiotic more effective. In contrast, ciprofloxacin‐tolerant mutants display metabolic repression and reduced ATP levels, leading to decreased antibiotic uptake and lower ROS production, which diminishes antibiotic efficacy. It is proposed that additional mechanisms, for example, ATP‐dependent modulation of antibiotic target activity, may also contribute to ciprofloxacin persistence.

Cellular ATP levels directly reflect metabolic activity. In this context, it is proposed that reduced ATP levels not only diminish the activity of antibiotic targets^15^, but also decrease bacterial uptake of antibiotics across the cytoplasmic membrane, a process that is typically energy‐dependent and transporter‐mediated. Furthermore, downstream lethal effects of bactericidal antibiotics, including the generation of ROS, partially depend on bacterial metabolic activity^65^, ^89^. In persister cells, metabolic activity is minimized due to reduced energy availability, enabling survival through mechanisms such as decreased target activity, inefficient antibiotic uptake, and attenuated downstream antibiotic‐induced damage, as summarized in the proposed model (Figure 8). Consistent with this framework, ciprofloxacin‐sensitive mutants showed higher levels of ciprofloxacin uptake and ROS accumulation, whereas ciprofloxacin‐tolerant mutants displayed lower levels of both parameters relative to wild‐type cells (Figure 7A–C).

Nevertheless, the efficacy of bactericidal antibiotics is contingent upon bacterial metabolic activity, and the degree of this requirement varies^70^, and it remains unclear whether tolerance to weakly metabolism‐dependent antibiotics, for example, polymyxin E and mitomycin C, is strictly governed by ATP levels. In this regard, Δ*atpC* and Δ*ruvC*, as ciprofloxacin‐sensitive mutants, were found to be more sensitive to polymyxin E, whereas Δ*nadB*, a ciprofloxacin‐tolerant mutant, showed increased tolerance (Figure S15A–D). In contrast, Δ*metR* largely displayed tolerance levels comparable to those of the wild type (Figure S15E). Although not exhaustive, these findings suggest that polymyxin E tolerance may also partially conform to the low ATP paradigm. Supporting this interpretation, ATP reduction by CCCP or arsenate increased polymyxin E tolerance in both wild‐type cells and sensitive mutants (Δ*atpC* and Δ*ruvC*) (Figure S15F), whereas ATP elevation through nutrient‐rich LB medium decreased polymyxin E tolerance in wild‐type and tolerant mutants (Δ*metR* and Δ*nadB*) (Figure S15G). Given that polymyxin E kills Gram‐negative bacteria by disrupting the outer membrane through displacement of divalent cations, it is hypothesized that cells with higher ATP levels may more effectively regenerate lipopolysaccharide and maintain membrane integrity, thereby increasing susceptibility. Conversely, cells with reduced ATP levels may show impaired membrane repair capacity, resulting in enhanced tolerance.

However, observations that challenge the general applicability of this paradigm should be noted. Specifically, Δ*atpC*, Δ*atpF*, Δ*ruvC*, and Δ*rnr* mutants showed elevated ATP levels (Figures 4C and 5A) but displayed tolerance to ampicillin and gentamicin comparable to that of wild‐type cells (Figures 2D and 4E). These findings underscore the complexity of persister formation and antibiotic tolerance, which clearly depend on the class of bactericidal antibiotic used. Accordingly, the low ATP–high antibiotic tolerance paradigm does not universally account for all aspects of persister biology. Moreover, the differential effects of arsenate treatment on ciprofloxacin tolerance in stationary‐phase Δ*atpC*/Δ*atpF* mutants versus wild‐type cells (Figure 5F), together with differences in ATP modulation (Figure 5G,H), suggest the existence of a threshold in ATP reduction that is required to significantly enhance persister tolerance. An additional unresolved question is whether other mutants identified in this study conform to the low ATP–high tolerance paradigm. For example, PhnH utilizes ATP as a substrate for the biosynthesis of α‐d‐ribose 1‐methylphosphonate 5‐triphosphate, suggesting that the ciprofloxacin‐sensitive Δ*phnH* mutant may possess elevated ATP levels. Similarly, YfgL (BamB) is a key component in outer membrane protein biogenesis, and its absence may disrupt membrane integrity, thereby altering antibiotic and nutrient uptake and ultimately influencing intracellular ATP levels and ciprofloxacin tolerance.

### Supplement of abundant nutrients is a clinically relevant strategy for anti‐persister chemotherapy

Under the low ATP–high antibiotic tolerance paradigm, modulation of nutrient abundance represents one of the simplest strategies to alter the antibiotic tolerance of persister cells by regulating intracellular ATP levels and metabolic activity. It is therefore not surprising that supplementation with specific metabolites, including amino acids and carbon sources, has been reported to markedly enhance the efficacy of antibiotics against persister cells^40^, ^49^, ^90^, ^91^, ^92^, ^93^, ^94^, ^95^. Consistent with these observations, Tenson and colleagues reported that resuscitation of stationary‐phase cultures in different fresh media resulted in markedly different persister frequencies^43^. In the present study, replacement of M9 glucose medium with nutrient‐rich LB medium was shown to significantly increase the efficacy of ciprofloxacin against *E. coli* wild‐type cells, as well as ciprofloxacin‐sensitive and ciprofloxacin‐tolerant mutants (Figure 6).

During resuscitation, lag‐phase duration varies among individual cells within a population, likely due to differences in dormancy depth^44^, ^96^ and/or single‐cell lag‐time distributions^45^, ^46^. As a result, growing and non‐growing cells coexist. Acceleration of resuscitation in non‐growing cells and enhancement of growth in growing cells through supplementation with nutrient‐rich media increase metabolic activity, thereby improving the efficacy of bactericidal antibiotics. This strategy has been demonstrated to be effective in eradicating persister infections in animal models^44^, ^90^, ^94^, and its potential for clinical antipersister therapy warrants further exploration. Nevertheless, this approach may be limited in the treatment of biofilm‐associated infections, in which growing cells and non‐growing persisters coexist^97^, as penetration of supplied nutrients into the biofilm interior, where non‐growing cells predominantly reside, is likely restricted by the outer biofilm layers.

In conclusion, this study demonstrates a pivotal role of intracellular ATP levels in determining ciprofloxacin tolerance among genetically diverse *E. coli* persisters, conceivably through modulation of antibiotic uptake and ROS‐mediated killing. A genome‐wide screen identified numerous persistence‐related genes and consistently revealed an inverse correlation between ATP levels and ciprofloxacin tolerance. The apparent universality of this mechanism suggests that modulation of bacterial metabolic states, rather than targeting individual genetic determinants, represents a promising strategy for the treatment of persistent bacterial infections.

## MATERIALS AND METHODS

### Bacterial strains and plasmids

The *E. coli* Keio and ASKA collections were obtained from the National Institute of Genetics, Japan^29^, ^48^. Single‐gene knockout strains (Δ*fis*, Δ*recN*, Δ*holC*, Δ*nfo*, Δ*rnr*, Δ*ruvA*, Δ*ruvC*, Δ*atpC*, Δ*atpF*, Δ*metR*, Δ*metE*, Δ*leuB*, Δ*leuL*, Δ*nadB*, Δ*nadC*, and Δ*recF*) were derived from the Keio collection. Plasmids derived from pCA24N for expression of Fis, RecN, HolC, Nfo, Rnr, RuvA, RuvC, AtpC, AtpF, MetR, NadB, and NadC were obtained from the ASKA collection. The plasmid pET28a‐*leuB* was constructed using conventional PCR‐based cloning and plasmid amplification. Plasmids pCA24N‐*ruvC* carrying point mutations were constructed using the Blunting Kination Ligation Kit (Takara Bio), according to the manufacturer's instructions.

### Screen for ciprofloxacin‐tolerant mutants using the Keio collection

Persisters were generated in stationary‐phase cultures and subsequently resuscitated in fresh medium for antibiotic treatment, following previously reported protocols^19^, ^43^. Briefly, 2 μl of each single‐knockout strain from the Keio collection (stored at −80°C) was diluted into 200 μl of LB medium in 96‐well plates and incubated overnight (37°C, 300 rpm). Cultures were then transferred at a 1:100 ratio into 200 μl of M9 medium supplemented with 0.5% glucose and incubated for 24 h (37°C, 300 rpm). Starvation‐induced persisters in the stationary phase were diluted 20‐fold into M9 glucose medium containing 2.5 μg/ml ciprofloxacin and incubated (37°C, 300 rpm) for 3 and 5 h to identify ciprofloxacin‐sensitive and ciprofloxacin‐tolerant mutants, respectively. Aliquots (4 μl) of 10‐fold diluted cultures were spotted onto LB agar plates for survival assays. Kanamycin (50 μg/ml) was present throughout all experiments. Δ*fis* ;(a ciprofloxacin‐sensitive mutant) and the wild‐type strain BW25113 were included in empty wells of each 96‐well plate for comparison. Re‐screening of candidates from the initial screen was performed under identical conditions, and the final mutants were validated by PCR‐based DNA sequencing using the primers listed in Table S1.

### Functional classification and interaction analysis of ciprofloxacin tolerance‐related genes

Functional classification of target genes was assigned based on annotations in the UniProt database (http://www.uniprot.org/) (Table 1). Protein–protein interaction networks were analyzed using the STRING database (version 12.0), with the species specified as *E. coli* K‐12 MG1655.

### Antibiotic tolerance assays under different conditions

Tolerance of mutants to additional bactericidal antibiotics was assessed using the procedures described above. Ampicillin (200 μg/ml), gentamicin (7.5 μg/ml), and ofloxacin (2.5 μg/ml) were added to the resuscitation medium, with treatment durations of 6, 3, and 5 h, respectively. Where indicated, specific metabolites (25 or 50 μg/ml methionine, 20 μg/ml leucine, 1 or 5 μg/ml NAD⁺, or 0.2% or 0.5% casamino acids) or ATP inhibitors (10 mM arsenate and 20 μM carbonyl cyanide *m*‐chlorophenyl hydrazone [CCCP]) were added to the resuscitation medium. LB medium was used at varying proportions to replace M9 glucose medium during resuscitation. Complementary gene expression was achieved by 20‐fold dilution of stationary‐phase cultures into IPTG‐containing fresh M9 glucose medium, followed by addition of 2.5 μg/ml ciprofloxacin after 1 h. Treated cells were washed once with PBS (0.27 g/l KH₂PO₄, 1.42 g/l Na₂HPO₄, 8 g/l NaCl, and 0.2 g/l KCl, pH 7.4), and 4 μl of 10‐fold serially diluted suspensions were spot‐plated onto LB agar plates for survival analysis.

### SDS‐PAGE and immunoblotting analysis

Cell lysates were separated by 10% or 15% SDS‐PAGE. His‐tagged proteins were detected using mouse anti‐His‐tag monoclonal antibodies (1:5000 dilution; ABclonal) as primary antibodies and alkaline phosphatase‐conjugated goat anti‐mouse IgG (H + L) antibodies (1:5000 dilution; Proteintech) as secondary antibodies. Protein bands were visualized using a BCIP/NBT chromogen kit (Solarbio).

### ATP assay

Intracellular ATP concentrations were quantified using a luciferase‐based ATP assay kit (S0026B; Beyotime Biotechnology), following the manufacturer's protocol. Briefly, cells were lysed, and lysates were centrifuged at 12,000*g* for 5 min at 4°C to remove debris. Supernatants were mixed with the working solution at a 1:1 ratio, and luminescence was immediately measured using a FLUOstar Omega microplate reader.

### Antibiotic uptake assay

Stationary‐phase wild‐type and mutant *E. coli* cells were normalized to equal cell densities and diluted into fresh M9 glucose medium containing 2.5 μg/ml ciprofloxacin. Cultures were incubated for 3 or 5 h, followed by lysozyme digestion for 2 h. Cells were subjected to three freeze–thaw cycles and heat‐denatured at 90°C for 10 min, as previously reported^73^, ^74^. After centrifugation, supernatants were analyzed for ciprofloxacin concentration using a commercial kit (ml036108; mlBio Inc.), according to the manufacturer's instructions.

### ROS assay

Intracellular superoxide levels were monitored using dihydroethidium (DHE), as previously described^98^. Stationary‐phase *E. coli* cells were diluted 20‐fold into M9 glucose medium containing 2.5 μg/ml ciprofloxacin and 100 μM DHE, followed by incubation for 1, 3, or 5 h. Cells were then subjected to flow cytometric analysis using a CytoFLEX instrument (Beckman Coulter).

### Statistical analysis

Colonies on LB agar plates were counted to determine colony‐forming units (CFUs). Cell densities were calculated based on dilution factors and plated volumes. Quantitative data, calculated using GraphPad Prism 9.0, are presented as mean ± SD from three replicates of one experiment; all experiments were independently repeated at least three times. Linear regression analysis of bacterial survival and ATP levels was performed using GraphPad Prism 9.0, and correlation analyses were conducted using SPSS 26.0. Statistical significance was assessed using two‐tailed tests.

## AUTHOR CONTRIBUTIONS

**Zhenfang Mei**: Data curation; validation; investigation; writing—original draft. **Yawen Cai**: Data curation. **Jianfeng Huang**: Investigation. **Kedong Zhao**: Data curation. **Zuqin Zhang**: Conceptualization. **Dandan Yu**: Data curation. **Shiyan Lu**: Data curation. **Zeying Lai**: Data curation. **Thomas K Wood**: Writing—review and editing. **Xinmiao Fu**: Funding acquisition; supervision; project administration; writing—original draft; writing—review and editing.

## ETHICS STATEMENT

No animal or human subjects were involved in this study.

## CONFLICT OF INTEREST

The authors declare no conflicts of interest.

## Supporting information

Supplemental figure.

## Data Availability

All data generated or analyzed during this study are included in the Supporting Information file.
