# Supplementary material for: Genome‐wide screen reveals a universal role of ATP in ciprofloxacin tolerance among genetically distinct Escherichia coli persisters
Source: mLife. 2026 Mar 19;5(2):180–98. doi: 10.1002/mlf2.70072 (PMC13131330; doi:10.1002/mlf2.70072)
Supplement: Supplementary file 1 — Supplemental figure. [file MLF2-5-180-s001.pdf]

Supporting Information File

Genome-wide screen reveals a universal role of ATP in ciprofloxacin tolerance among genetically distinct Escherichia coli persisters

Zhenfang Mei [1], Yawen Cai [1, 2], Jianfeng Huang [1], Kedong Zhao [1, 2], Zuqin Zhang [1, 2], Dandan Yu [1], Shiyan Lu [1], Zeying Lai [1], Thomas K. Wood [3] and Xinmiao Fu [1,2, #]

[1] Provincial University Key Laboratory of Cellular Stress Response and Metabolic Regulation, College of Life Sciences, Fujian Normal University, Fuzhou, China
[2] Institute of Precision Medicine, Fujian Provincial Hospital, Fuzhou University Affiliated Provincial Hospital, Fuzhou, China
[3] Department of Chemical Engineering, Pennsylvania State University, University Park, PA, USA

[#] To whom correspondence should be addressed to Professor Xinmiao Fu (xmfu@fjnu.edu.cn)

Results

Table S1 Oligonucleotides for mutant confirmation

Mutation	Forward Primer (5'→3')	Reverse Primer (5'→3')
Δ*fis*	TCAACGCCATTGAGGATG	GTCGGTTCACATCCTGTTCTC
Δ*rnr*	CAGAGATGACAACGGAGG	ATGTAGGCCGGATAAGGC
Δ*uvrD*	GATCTTACATGTTGGGGAC	GCGAAACTGAACGTTGAG
Δ*nfo*	GTGATTCAAAGCGTCATCGC	CAGTATTCCTGCTCGGTGAG
Δ*ruvA*	CTGCTTCAATCATCCTTTACC	ATTACGCAGGAGCGTCAT
Δ*ruvC*	TGGGTTCTATTCCGTGGGA	TCGCAGCGACTCTCTGAT
Δ*holC*	GAAGGCGTGACCCATATGC	TGCGGTGTATACCTGATGC
Δ*recG*	GCTACTATGCAGGCTGCA	ATGGCGGTCTTCTCACTG
Δ*recN*	AGTTGCGACAGCCAGATA	GGTCAGATAGTTCCCCTG
Δ*yfgL*	CAACGCACGCTATATTCGCG	CGTAGTGCATGGGAAGCAG

Δ*rfaH*	AACGTATTGCGCACTGG	CGTTCATCTTTGCGATGC
Δ*rimK*	ATCAACCGCTGGATAAAG	CAGGATCGTGTTATCGAT
Δ*xseA*	TTCGTCAGCTGGGTGCTGA	GTGCTTGATCAGGATAACGGCC
Δ*atpC*	CTGTTTCCAGACTGGCTT	GCCTTAATCGGAGGGTGA
Δ*atpF*	GGCGAGCTACCGTAATAA	CTAAATAGAGGCATTGTGC
Δ*atpG*	ACCTCAAGAGCATCGTACA	CCTCGATTCCTTCAAAGCA
Δ*ymfB*	TGAACTTCAAGGCGGCGTA	GGTGAATGGCGAGAAGTGA
Δ*pdxJ*	ATGCGAGCGATCTCCACA	TAAGCCGTATCTTGGCGG
Δ*coaE*	GTTCAAAAAGGACCTGGGT	GTGAGCGAACGTTTCATCA
Δ*gpmM(gpmI)*	CTCTTGAGTATGAGGTTGTC	CGTGTCATGGTATTAATCGC
Δ*pncA*	TGACGCCGGATGATTAAGGA	TACAGAATTGTAGGCCAGCG
Δ*tktA*	CTTGTCCGCAAACGGACATATC	CTTCATCATCCGATCTGGAGTCA
Δ*rffC*	GATGACGAGGGTGTGAAG	TCGAGTTCGGTTCCCAC
Δ*aroD*	AGGCGCAACAAGCTGGT	TAGCGCACAGAGACTCACGA
Δ*emtA*	TCACCTCAACGGCGATTCCA	GACAAAGTGCGCGACTGA
Δ*ycdR*	CCTCATCATCAGTTCACCAG	GACCTTATGACGGTGACAGA
Δ*iscR*	TAGCACTCCGGCCTGATTC	GTCAGACTTGACCCTGCTATG
Δ*ycgE*	AGCTCCGGTACAAACGCTG	GTGTACAGGCGACGGAGAT
Δ*minC*	CGCTGGAGGTTGTCTTACC	AACATCATCGCGCGCTG
Δ*ftsP(sufI)*	GCCGGAACGAAAGAGAAATCG	AAGATCAGGTTCGTGAGCTGG
Δ*rppH(nudH)*	CAACGTCGATCATGATCGGC	TGTAATGCCCTGCAGAGAGTG
Δ*gfcC(ymcB)*	GCCAAAGTCTGATTGCGACGAA	CAGGTTCGTCAAAGTCGGCAA
Δ*ivY*	ATCGTGTTATCGCCAGGC	CGACGAAGCCGGTAAAGTT
Δ*phnH*	TCGGTCATCACGCGATCTA	TGACCGTATGGCACGCAT
Δ*yohG*	CTTCTTCATCTGAGTGCCAG	AACACACGGTAATCTCCGC
Δ*yegV*	CTGGATGCGGTAAATCAGCT	GAGTACAGCATTAGCCACTGTC
Δ*ycaL*	TAATGGTAGCGTCGCTTGTC	GGGCCATCAATGGTAATAACCG
Δ*ypjL*	TATGAAGCATCTCCAGCG	GATGGCTGAAGACTCTATC
Δ*ydfI*	GGCATGAACTAAGCGTGTGA	AATCCCTTCTCTTACCGGAGAG
Δ*ydhL*	GCTGAACCATAATCGCTTCCTC	GAGTGTAGTAGACCAGTCGGTCT
Δ*fkpB*	GATGCGATGCTGACGCATCT	CAACAATGCTGATAGCGCGGT
Δ*pcnB*	GAGTAGCTCTTCAGGTGCAAG	GACACTACCGAGGTGTACT
Δ*ynfC*	GGGTCAGACTCTCCTGAA	CACAGCAGGTTGAGAGTT
Δ*mbhA*	CGTCAATCGCCATCTGT	GCAGTCCATATCCACATG
Δ*ynjB*	TCGATGATGCCCTGCGTA	TAAATCACCGCCACCATC
Δ*yhcD*	CCAGCCGATATGGTTATCGC	AAGTTCAGCAGCAGGCATC
Δ*recF*	CATCCGTGTCGCCGATAT	GGCTTATGTTGTCATGCC
Δ*metR*	CTGGGAAATGCGCAGAAAAT	CCGAGGGTGTGATTCAATATTG
Δ*metE*	GGATGTGTAAACATCTGGACGG	GCAGCGTATGCTGGAATGGTT
Δ*leuB*	CGTGAGCGTCGAACAATT	AGTTGCAACGCAAAGCTC
Δ*leuL*	GCCTGTAACGCCTGTTCAC	GTCAGGGTTGACATCCGTT

Δ*nadB*	CCGCTTCGATCCATGAGAGATC	AGCGATCGCTTAGTCATTCGTG
Δ*nadC*	GATGGAGCGGATAAATCTGTC	ATGATTCGTTAGCTATCTGGAG
Δ*glnE*	ACATCACCAACCACCATCACTC	AACCGTTCTGGTTGCACA
Δ*gshA*	GTGAAGCTATCTAACAACGGC	GCACATATGGTCACCATTAC
Δ*ygfj*	GACTAATGCCGATGGGTCAAC	CGGCTCGAAACTTAAGGGCT
Δ*sspA*	GATAGGGACGACGTGGTGT	GACTCACCACAATGGTCGC
Δ*glnP*	ATCGATATTGTGCAGCACCTGG	TGCGCGAGAACGGAACTTACA
pCA24N-*ruvC*(E66A)	CGCCATTGCACAAGTCTTTATGGC	AAATAATCAGGCTGGAACTGGGT
pCA24N-*ruvC*(D138A)	GGCAGCCGCCGATGCGCTG	GCCTGTGGATTAGCGGGCAGTTTC
pCA24N-*ruvC*(D141A)	CCGCAGCGCTGGCGATTGCTA	CGGCATCCGCCTGTGGATTAG

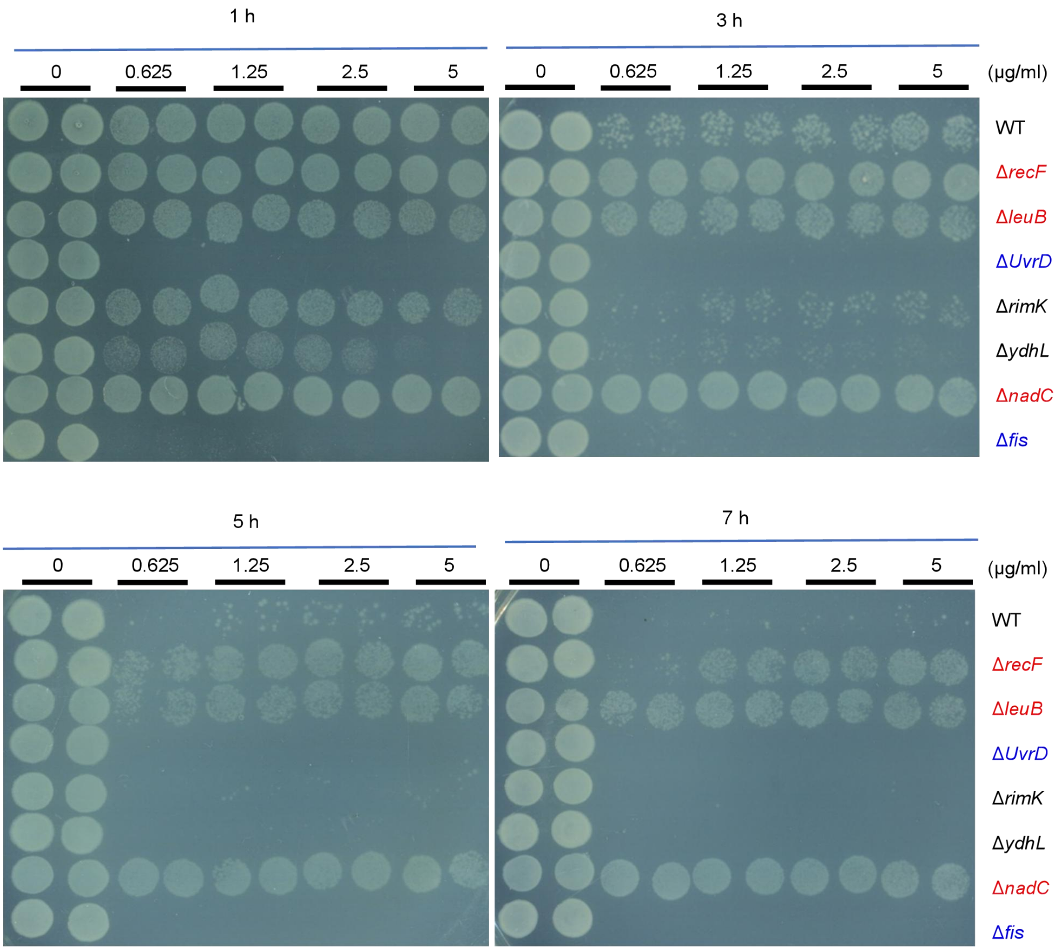

Fig. S1 Optimization of experimental conditions for screen of *ciprofloxacin-tolerant mutants*

Survival of indicated *E. coli* cells following ciprofloxacin treatment at indicated concentrations for varying length of time in M9 glucose medium. Cells were cultured

with M9 glucose medium in 96-well plates for 24 h before 20-fold diluted into fresh medium containing ciprofloxacin. Red texts represents ciprofloxacin-tolerant mutants, and blue texts represents ciprofloxacin-sensitive mutants.

(A)

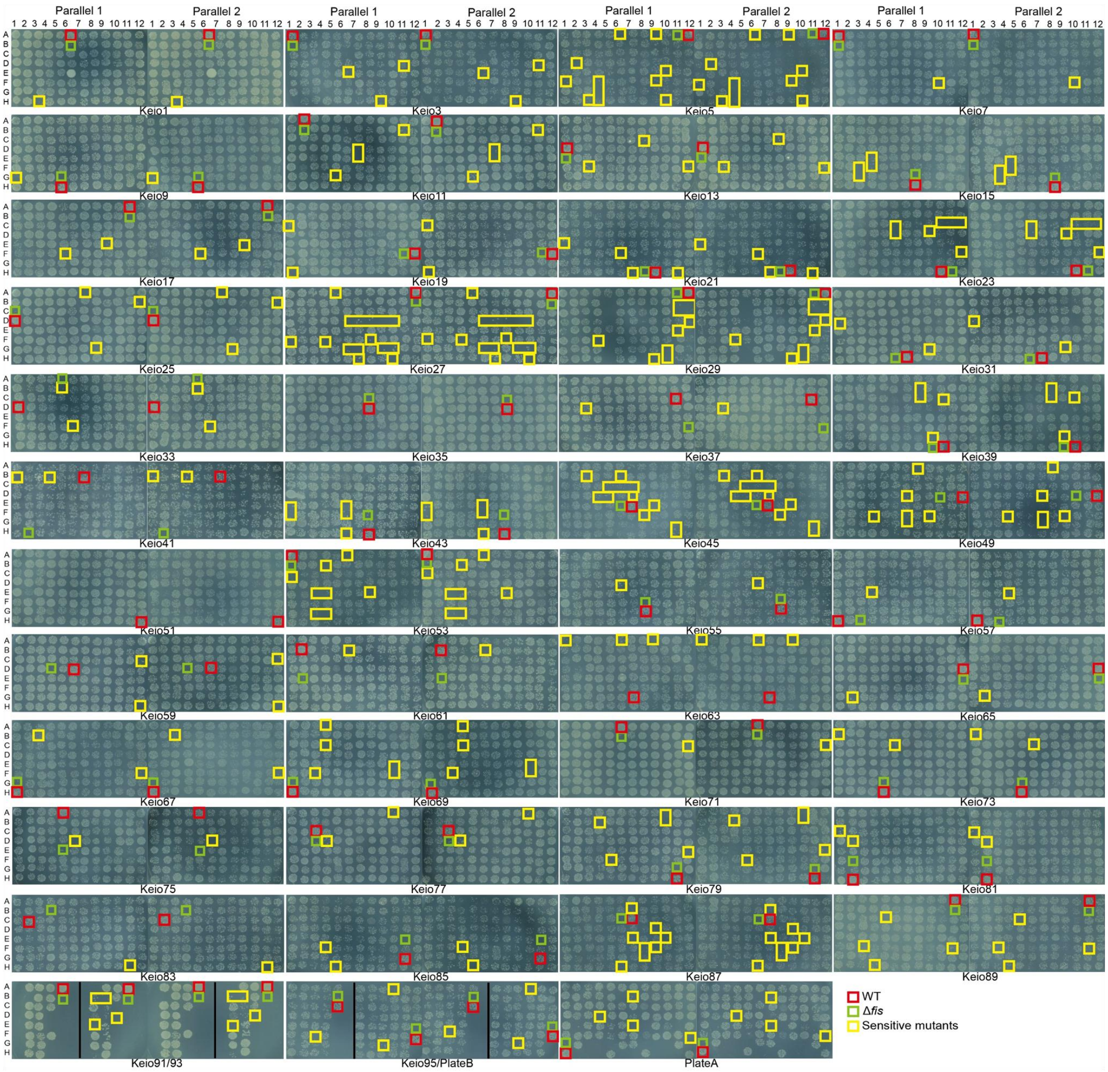

(B)

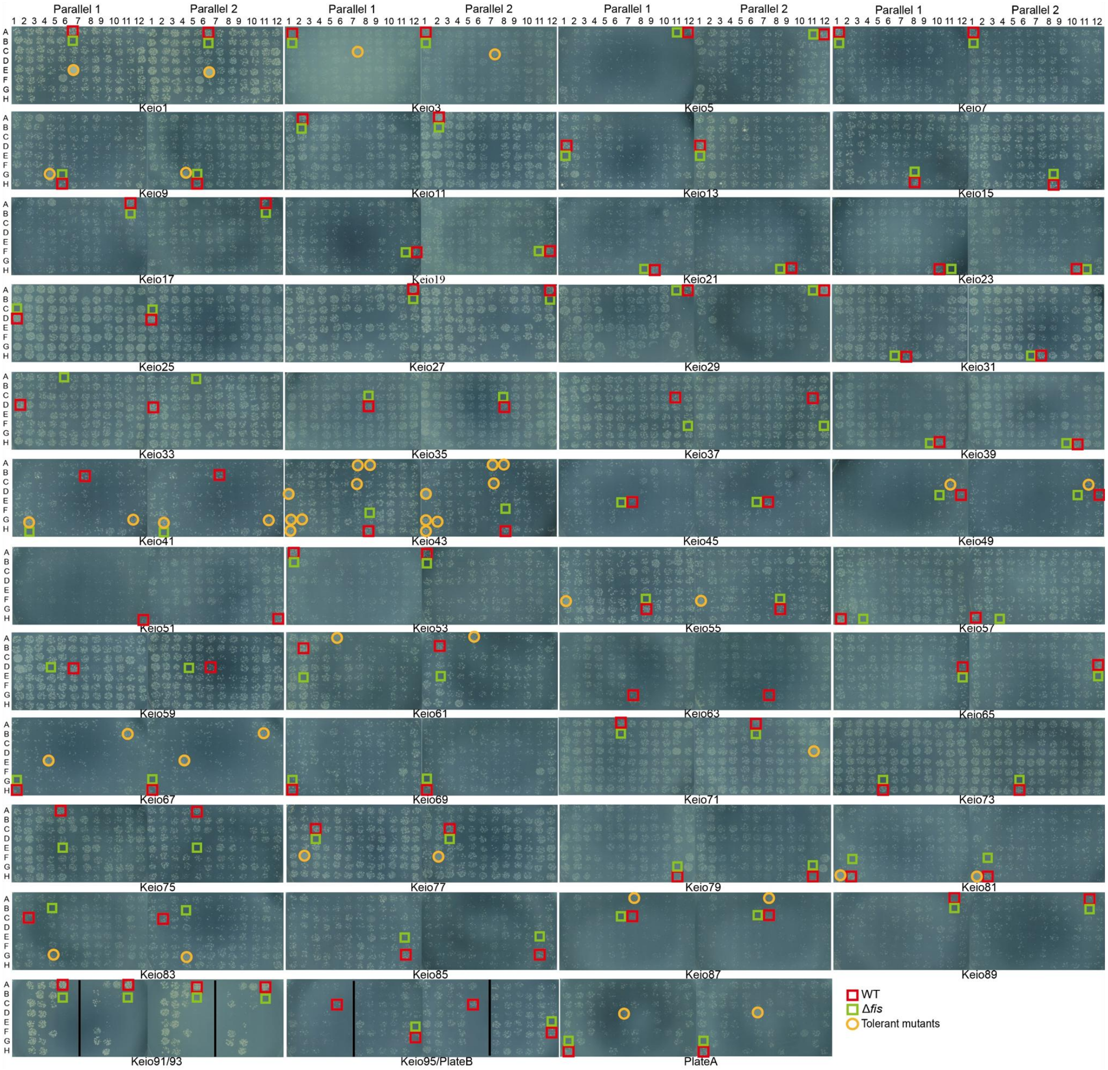

Fig. S2 Initial screen of *ciprofloxacin-tolerant mutants* from the Keio Collection

(**A, B**) Survival of *E. coli* Keio Collection after each strain was treated with 2.5 μg/mL ciprofloxacin for 3 h (panel A) and 5 h (panel B) in 96-well plates. Persister resuscitation and antibiotic killing experiments were replicated in parallel using two 96-well plates.

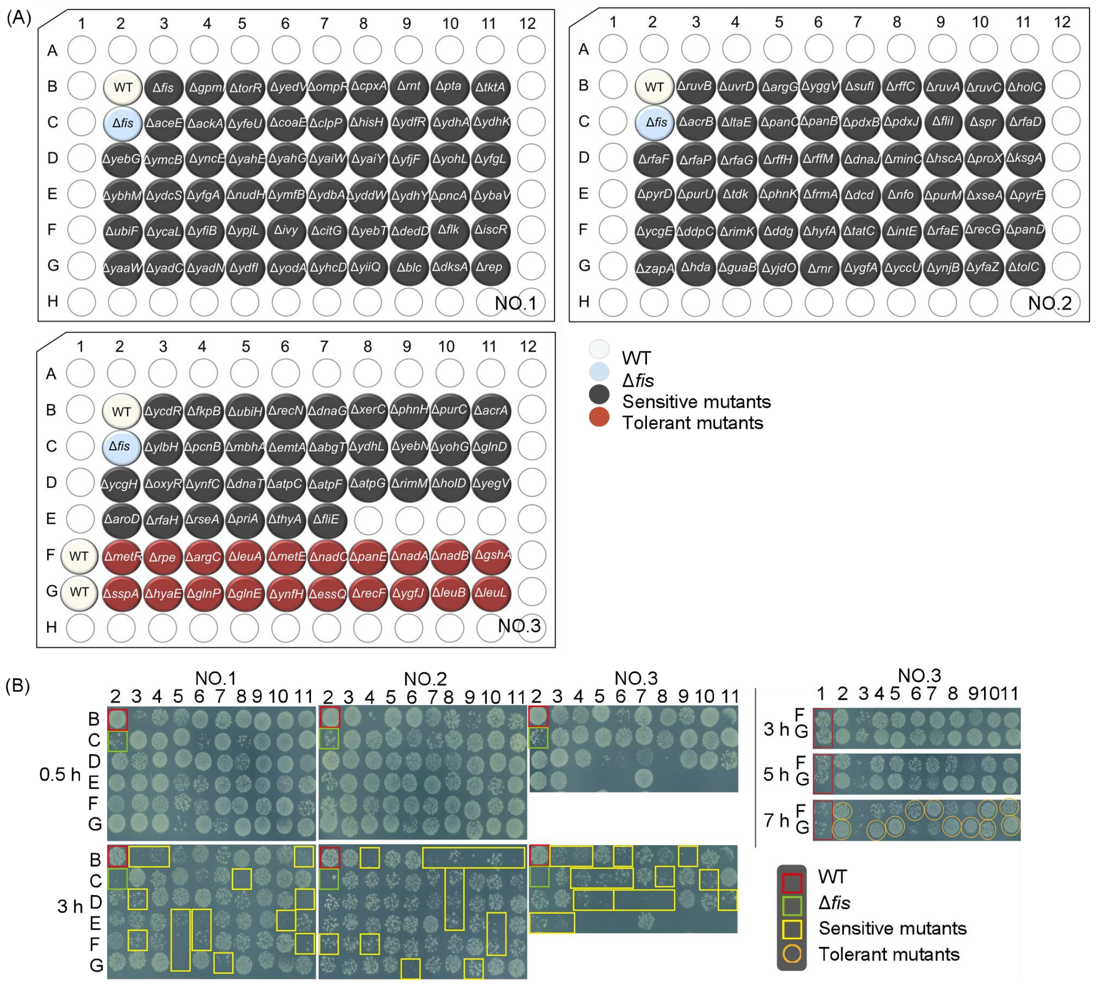

Fig. S3 Re-screen of the mutants obtained from initial screen

(**A**) Arrangement of 150 ciprofloxacin-sensitive mutants (colored in black) and 20 ciprofloxacin-tolerant mutants (colored in red) as isolated from initial screen as described in Fig. S2. (**B**) Survival of the mutants (panel A) after re-screen using the following conditions: treatment with 2.5 μg/mL ciprofloxacin for 0.5 h and 3 h (for sensitive mutants) and for 3 h, 5 h and 7 h (for tolerant mutants). 4 μL ten-fold diluted cultures were spotted on LB agar dish for cell survival assay.

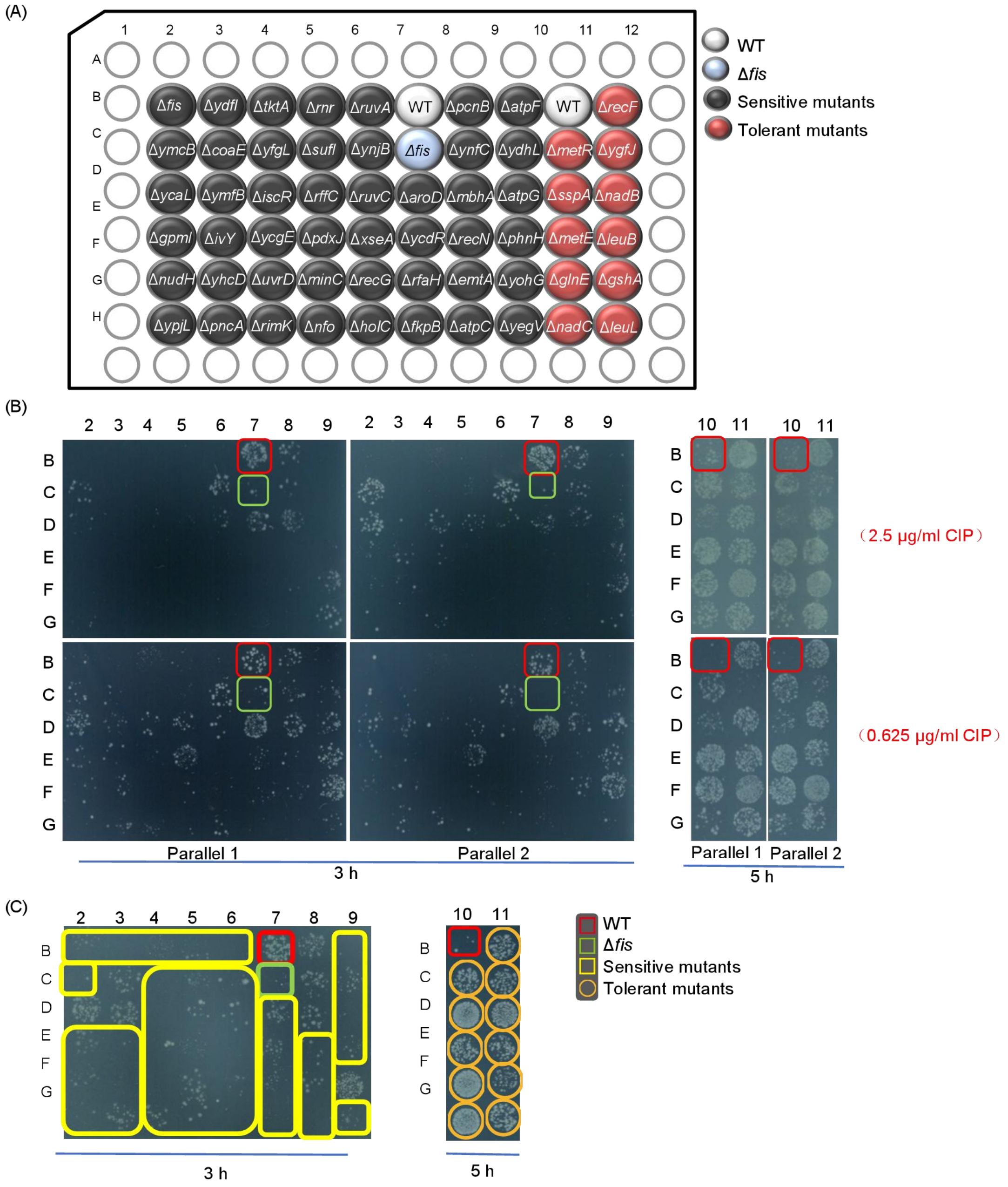

Fig. S4 Ofloxacin tolerance of *ciprofloxacin-tolerant mutants*

(**A**) Arrangement of 37 ciprofloxacin-sensitive mutants (colored in black) and 11 ciprofloxacin-tolerant mutants (colored in red) as isolated from screen as described in Fig. S3. (**B**) Survival of the mutants (panel A) following treatment with 2.5 µg/mL (upper part) or 0.625 µg/mL ciprofloxacin (lower part) for 3 h and 5 h towards sensitive and tolerant mutants, respectively. 4 µL ten-fold diluted cultures were spotted on LB agar dish for cell survival assay. (**C**) Survival of the ciprofloxacin-sensitive and ciprofloxacin-tolerant mutants following treatment with 2.5 µg/mL ofloxacin for 3 h (left part) and for 5 h (right part), respectively.

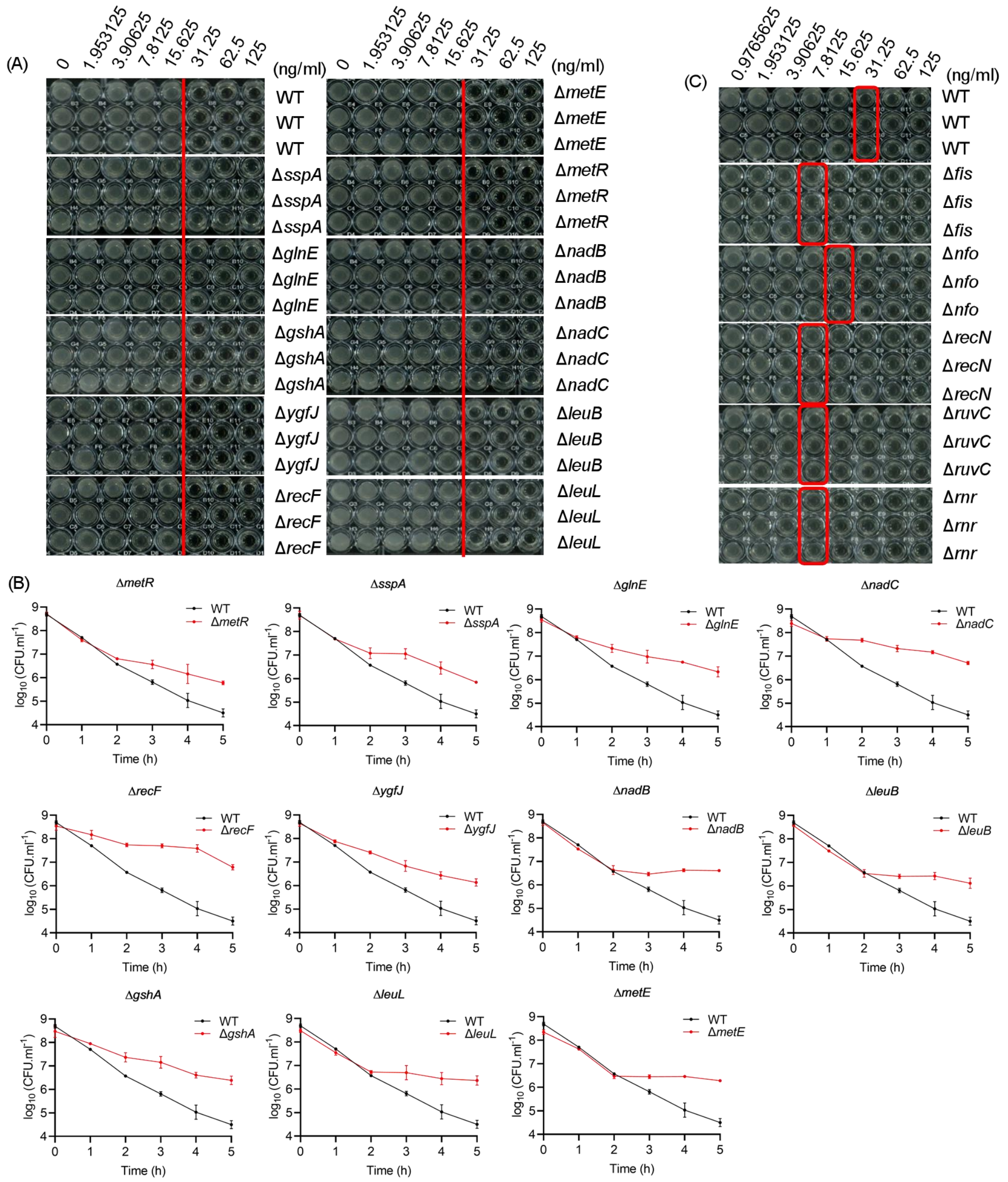

Fig. S5 Characterization of the ciprofloxacin tolerant mutants

(**A, C**) MIC assay results of the 11 ciprofloxacin-tolerant mutants (panel A) and 5 ciprofloxacin-sensitive mutants (panel C) in MHB medium by two-fold serial dilution of the antibiotic. (**B**) Time-dependent killing results of the 11 ciprofloxacin-tolerant mutants following treatment with 2.5 μg/mL ciprofloxacin during resuscitation.

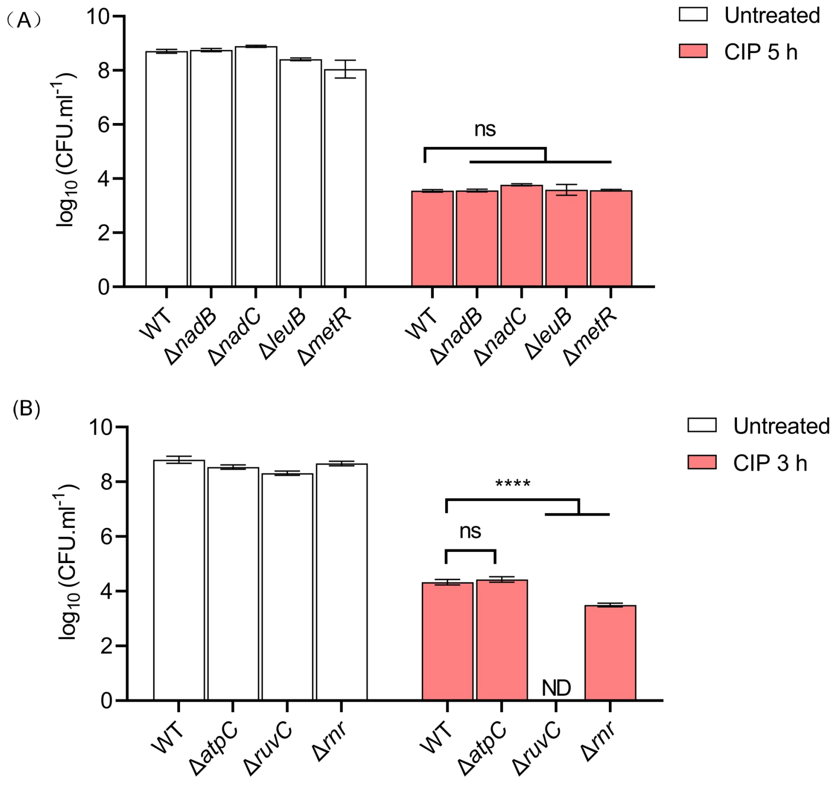

Fig. S6 Characterization of the ciprofloxacin tolerant mutants in exponential-phase

(**A**) Survival of tolerant mutant cells (Δ*nadB*, Δ*nadC*, Δ*leuB* and Δ*metR*) in exponential-phase following 5-h treatment with 2.5 µg/mL ciprofloxacin. Cells were cultured in M9 glucose medium to an OD_{600} of 0.15 before antibiotic treatment. (**B**) Survival of sensitive mutant cells (Δ*atpC*, Δ*ruvC* and Δ*rnr*) in exponential-phase following 3-h treatment with 2.5 µg/mL ciprofloxacin. ND: no CFU detectable on LB dishes. All experiments were conducted in triplicate. Error bars represent standard errors. Results of analysis of variance with Tukey's HSD post hoc tests are shown; ****$p < 0.0001$; ns, not significant.

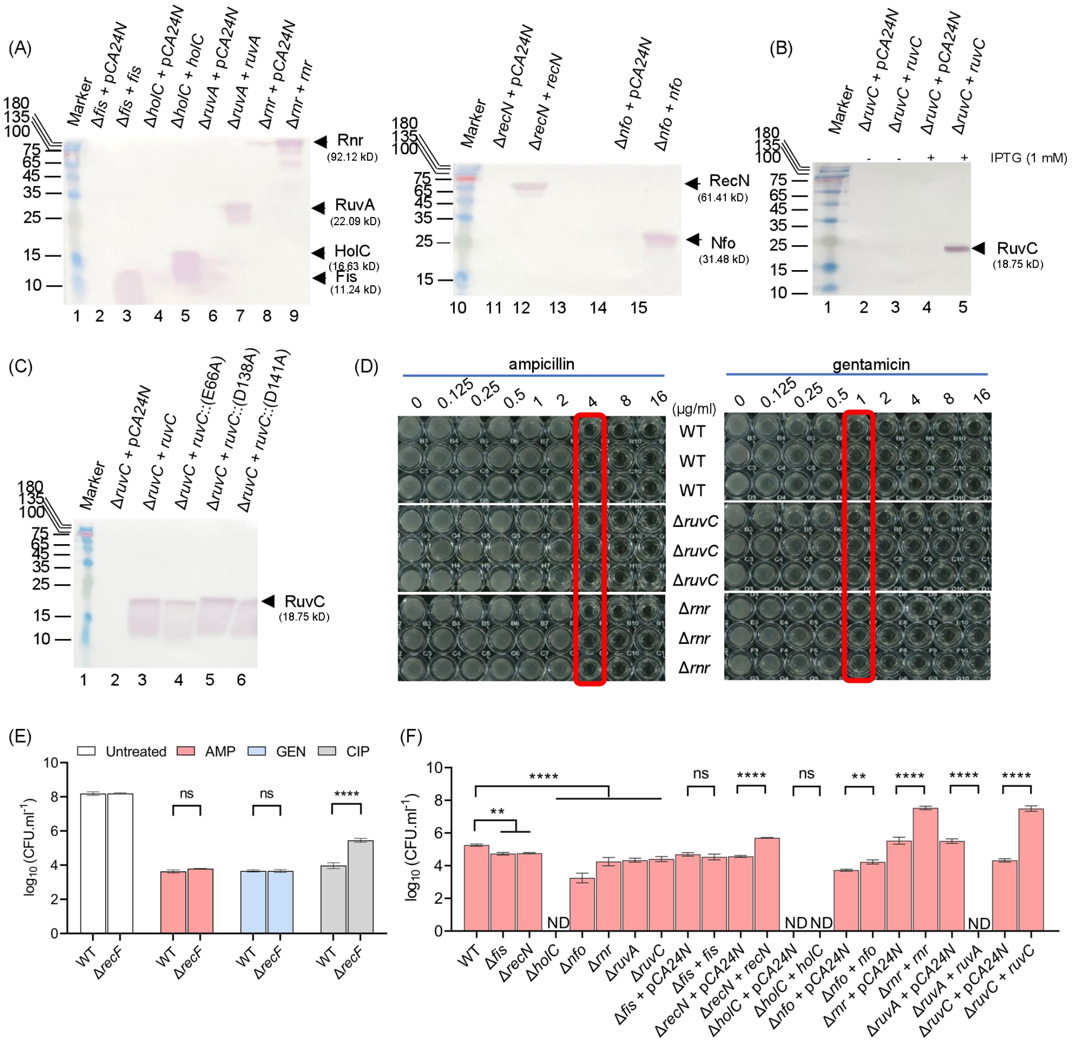

Fig. S7 Characterization of DNA repair-related mutants

(**A**) Immunoblotting results indicating the complementary expression of the indicated proteins in the corresponding single-knockout mutants using anti-His antibodies. Plasmids for protein expression were from ASKA collection. (**B, C**) Immunoblotting results indicating the expression of RuvC upon IPTG induction (panel B) and its enzymatic deficiency mutant proteins (panel C). (**D**) MIC assay results of Δ*ruvC* and Δ*rnr* mutants in MHB medium by two-fold serial dilution of ampicillin (left part) and gentamicin (right part). (**E**) Survival of wild type and Δ*recF* cells following treatment with 2.5 μg/mL ciprofloxacin for 5 h, with 200 μg/mL ampicillin for 6 h, or with 7.5 μg/mL gentamicin for 3 h. (**F**) Survival of indicated *E. coli* mutant cells following 3-h treatment with 2.5 μg/mL ciprofloxacin during resuscitation. ND: no CFU detectable on LB dishes. All experiments were conducted in triplicate. Error bars represent standard errors. Results of analysis of variance with Tukey's HSD post hoc tests are shown; **$p < 0.01$; ****$p < 0.0001$; ns, not significant.

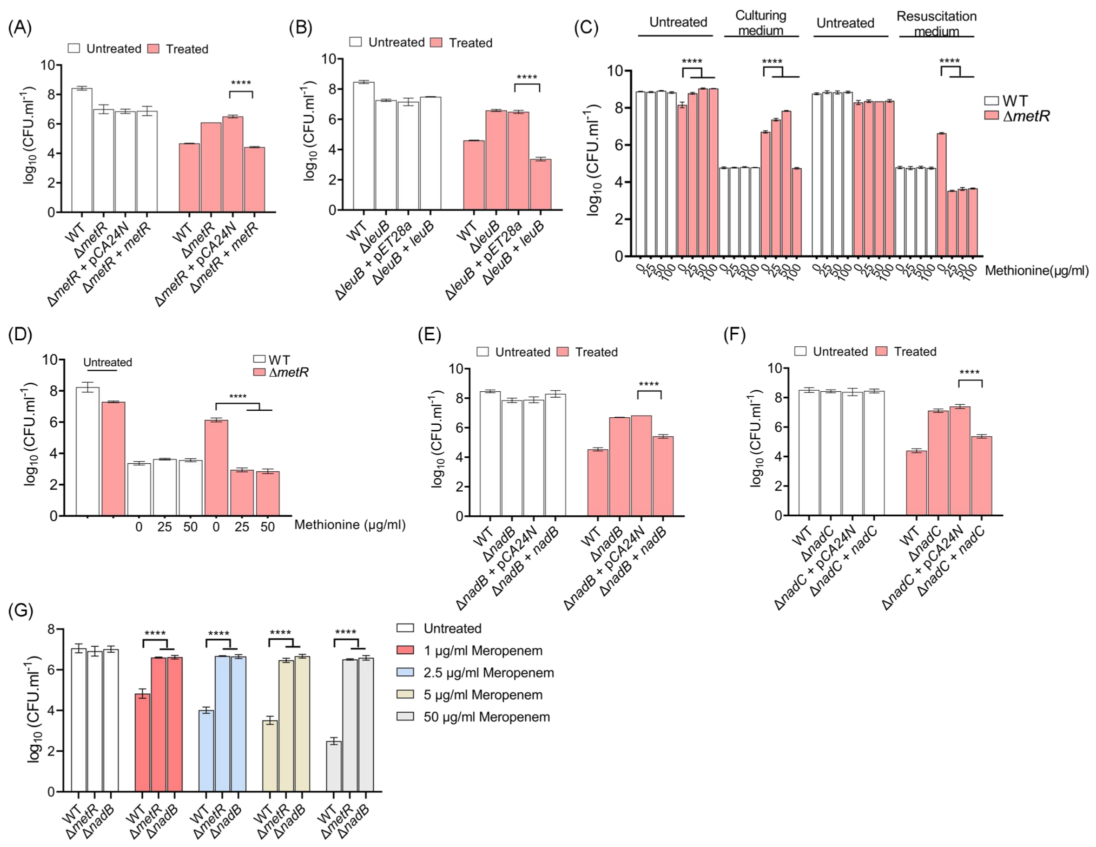

Fig. S8 Characterization of Δ*metR*, Δ*leuB*, Δ*nadB* and Δ*nadC*

(**A, B, E, F**) Survival *of* Δ*metR* (panel A), Δ*leuB* (panel B), Δ*nadB* (panel E) and Δ*nadC* (panel F) following 5-h treatment with 2.5 µg/mL ciprofloxacin during resuscitation upon the complementary expression of the corresponding protein. (**C**) Survival of Δ*metR* mutant cells following 5-h treatment with 2.5 µg/mL ciprofloxacin with 25, 50 or 100 µg/mL methionine being added in culturing medium or resuscitation medium. (**D**) Survival of Δ*metR* mutant cells following 5-h treatment with 7.5 µg/mL gentamicin during resuscitation in the presence of 25 or 50 µg/mL methionine. (**G**) Survival *of* Δ*metR* and Δ*nadB* cells following 5-h treatment with indicated concentrations of meropenem during resuscitation in M9 glucose medium. All experiments were conducted in triplicate. Error bars represent standard errors. Results of analysis of variance with Tukey's HSD post hoc tests are shown; ****$p < 0.0001$.

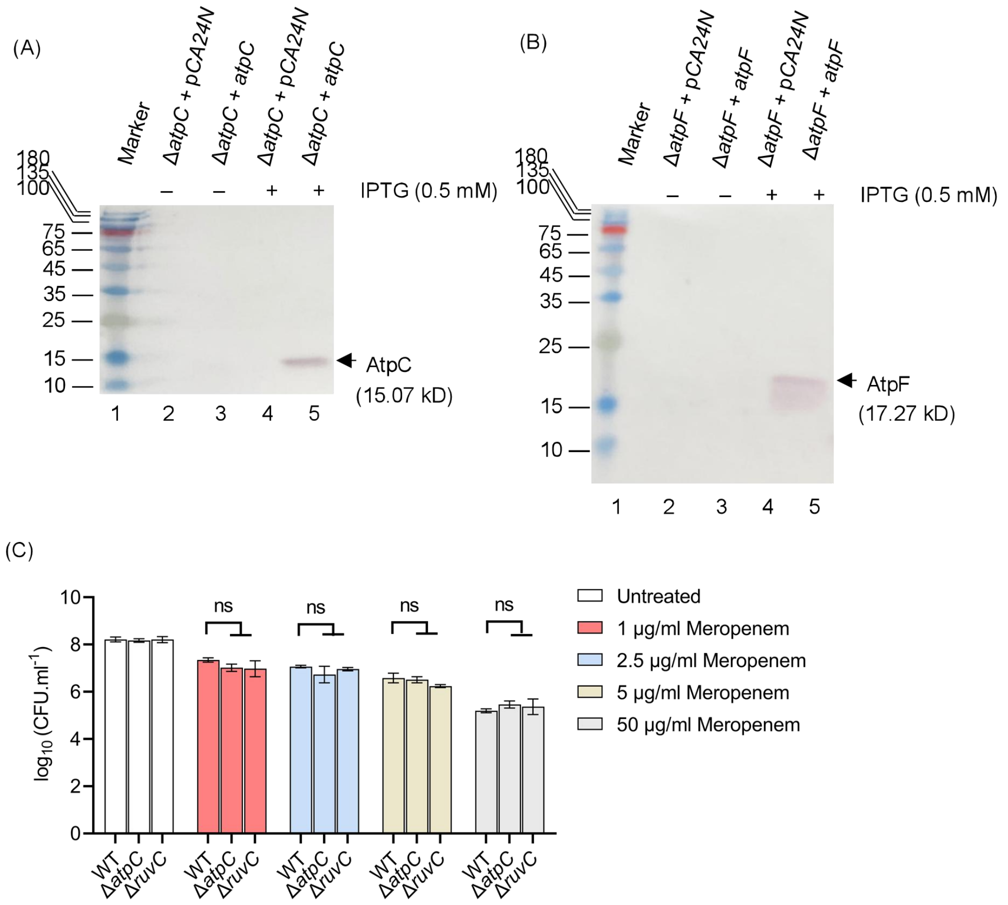

Fig. S9 Characterization of sensitive mutants

(**A, B**) Immunoblotting results indicating the IPTG-induced complementary expression of AtpC (panel A) and AtpF (panel B) in Δ*atpC* and Δ*atpF* cells, respectively. (**C**) Survival *of* Δ*atpC* and Δ*ruvC* cells following 3-h treatment with indicated concentrations of meropenem during resuscitation in M9 glucose medium. All experiments were conducted in triplicate. Error bars represent standard errors. Results of analysis of variance with Tukey's HSD post hoc tests are shown; ns, not significant.

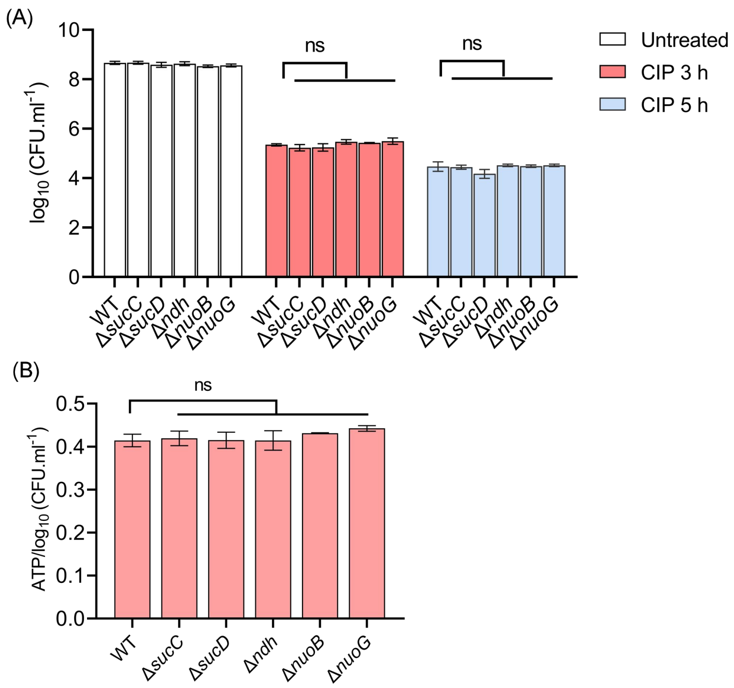

Fig. S10 Ciprofloxacin tolerance and ATP levels of typical mutant cells related to TCA cycle and respiratory chain

(**A**) Survival *of indicated mutant cells* following 3-h and 5-h treatment with 2.5 μg/mL ciprofloxacin during resuscitation in M9 glucose medium. (**B**) ATP levels of indicated mutant cells following 1-h resuscitation; cell density of these cells were adjusted to the same level prior to the resuscitation. All experiments were conducted in triplicate. Error bars represent standard errors. Results of analysis of variance with Tukey's HSD post hoc tests are shown; ns, not significant.

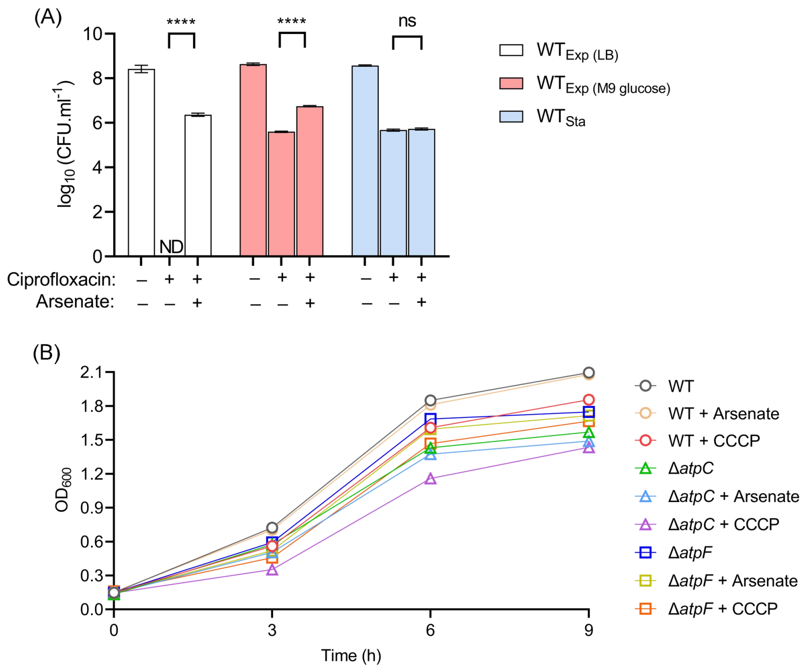

Fig. S11 Arsenate treatment increases the ciprofloxacin tolerance of *E. coli* cells in exponential-phase and inhibits cell growth during resuscitation

(**A**) Survival *of E. coli* wild type cells in exponential-phase in LB medium or M9 glucose medium following 3-h treatment with 2.5 μg/mL ciprofloxacin in the presence of 10 mM arsenate, which were directly added into LB cell cultures for treatment. For comparison, *E. coli* cells were cultured in M9 glucose medium into stationary-phase before 20-fold diluted into fresh medium containing ciprofloxacin and arsenate for resuscitation and treatment. (**B**) Cell growth curves of stationary-phase *E. coli* cells of indicated genotypes after 20-fold dilution into fresh M9 glucose medium containing 10 mM arsenate or 20 μM CCCP. ND: no CFU detectable on LB dishes. All experiments were conducted in triplicate. Error bars represent standard errors. Results of analysis of variance with Tukey's HSD post hoc tests are shown; ****$p < 0.0001$; ns, not significant.

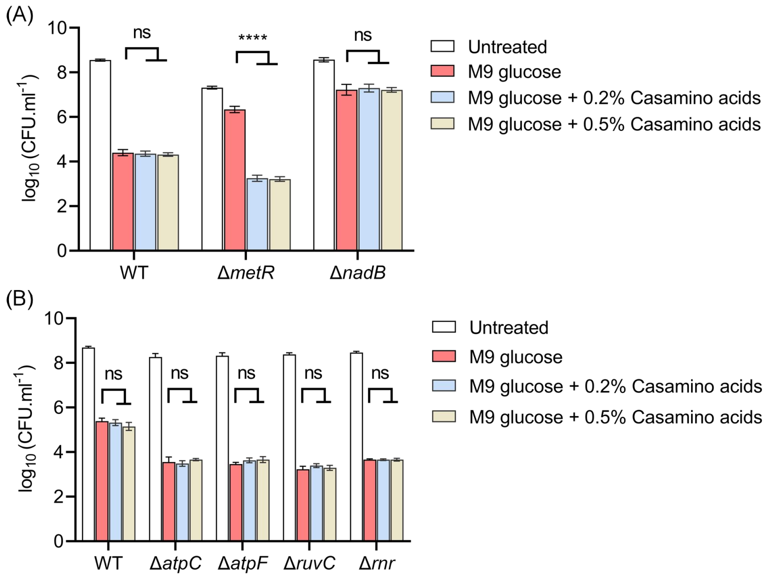

Fig. S12 Ciprofloxacin persistence of mutant cells upon the supply of casamino acids

(**A**) Survival of WT, Δ*metR* and Δ*nadB* mutant cells following 5-h treatment with 2.5 μg/mL ciprofloxacin during resuscitation in M9 glucose medium containing 0.2% or 0.5% casamino acids. (**B**) Survival of WT, Δ*atpC,* Δ*atpF,* Δ*ruvC* and Δ*rnr* mutant cells following 3-h treatment with 2.5 μg/mL ciprofloxacin during resuscitation in M9 glucose medium containing 0.2% or 0.5% casamino acids. All experiments were conducted in triplicate. Error bars represent standard errors. Results of analysis of variance with Tukey's HSD post hoc tests are shown; ****$p < 0.0001$; ns, not significant

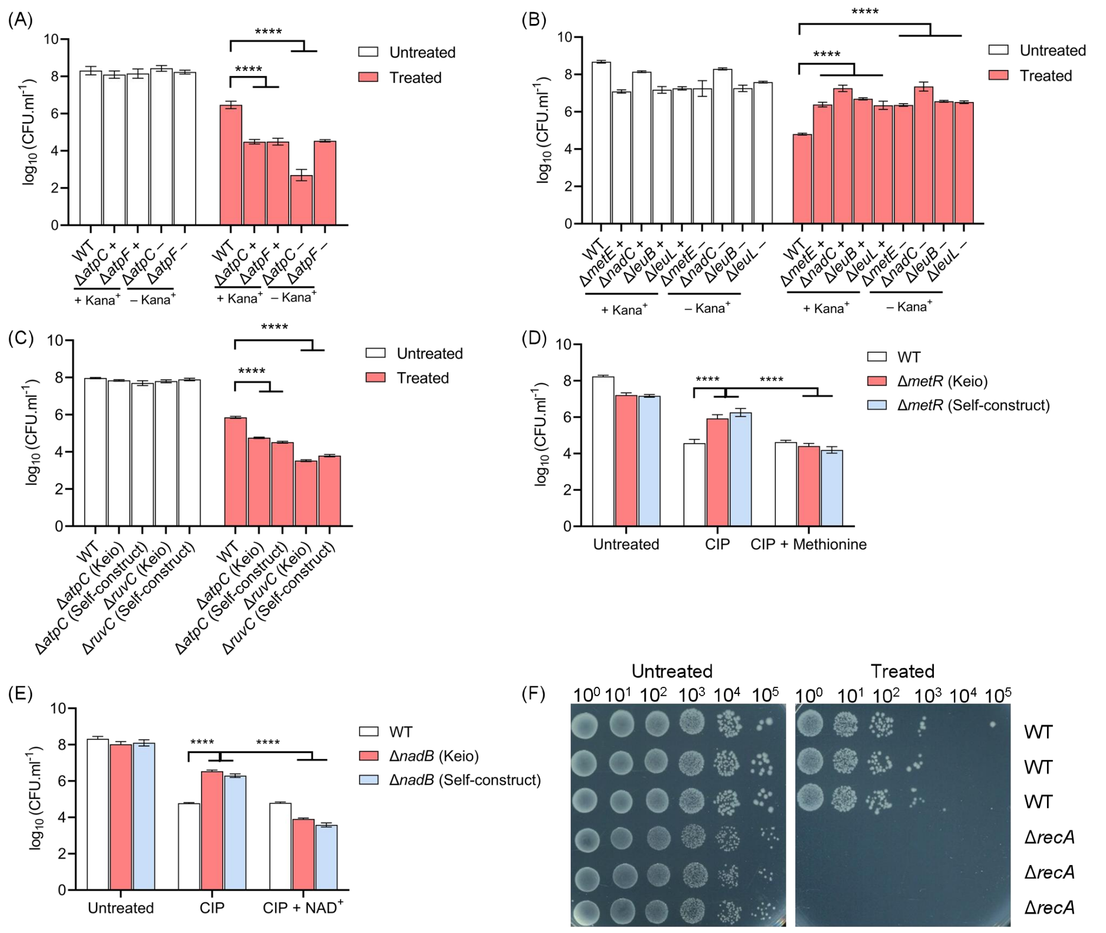

Fig. S13 Gene-deletion mutants cells after removal of the KAN-cassettes or self-constructed exhibited the same ciprofloxacin tolerance phenotypes as the corresponding mutants from the Keio collection

(**A, B**) Survival of Δ*atpC,* Δ*atpF,* Δ*metE,* Δ*nadC,* Δ*leuB* and Δ*leuL* mutant cells after removal of the KAN-cassettes (kanaymcin-resistant gene) following 3-h treatment (panel A for sensitive mutants) and 5-h (panel B for tolerant mutants) with 2.5 µg/mL ciprofloxacin during resuscitation. (**C, D, E**) Survival of Δ*atpC,* Δ*ruvC,* Δ*metR* and Δ*nadB* mutant cells following 3-h treatment (panel C for sensitive mutants) and 5-h (panel D and panel E for tolerant mutants) with 2.5 µg/mL ciprofloxacin during resuscitation; gene was deleted by ourselves using CRISPR-cas9. (**F**) Survival of wild type and Δ*recA* mutant cells following 3-h treatment with 2.5 µg/mL ciprofloxacin during resuscitation. All experiments were conducted in triplicate. Error bars represent standard errors. Results of analysis of variance with Tukey's HSD post hoc tests are shown; ****$p < 0.0001$.

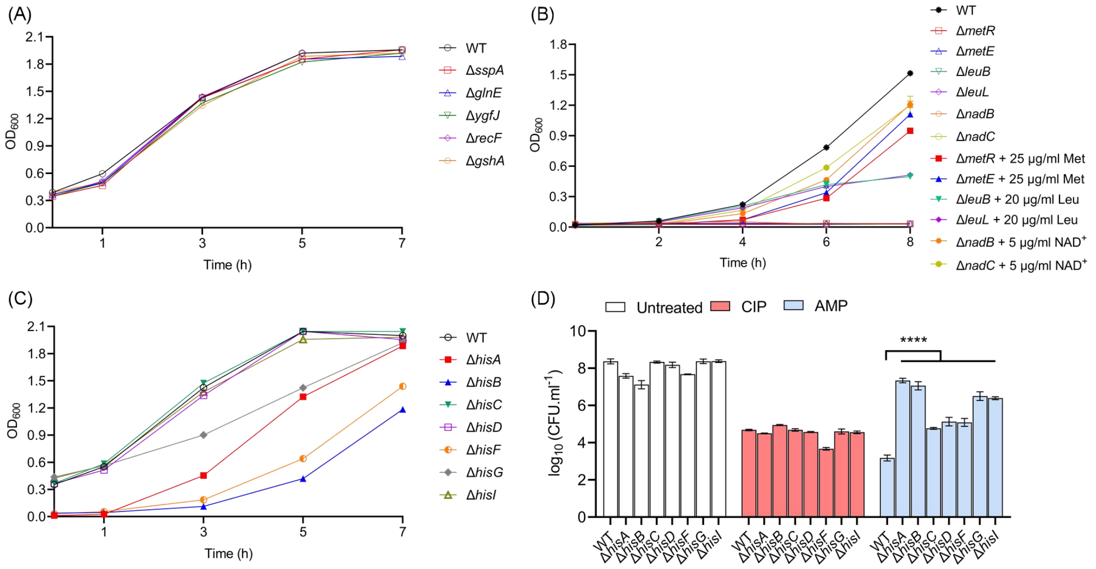

Fig. S14 No-growth phenotype is not sufficient for CIP persistence.

(**A, B, C**) Growth patterns of indicated mutants with or without supplementation of 25 μg/mL methionine (Met), 20 μg/mL leucine (Leu), or 5 μg/mL NAD⁺ in resuscitation medium. (**D**) Survival of Δ*hisA,* Δ*hisB,* Δ*hisC,* Δ*hisD,* Δ*hisF,* Δ*hisG* and Δ*hisI* mutant cells following treatment with 2.5 μg/mL ciprofloxacin for 5 h or with 200 μg/mL ampicillin for 6 h. All experiments were conducted in triplicate. Error bars represent standard errors. Results of analysis of variance with Tukey's HSD post hoc tests are shown; ****$p < 0.0001$.

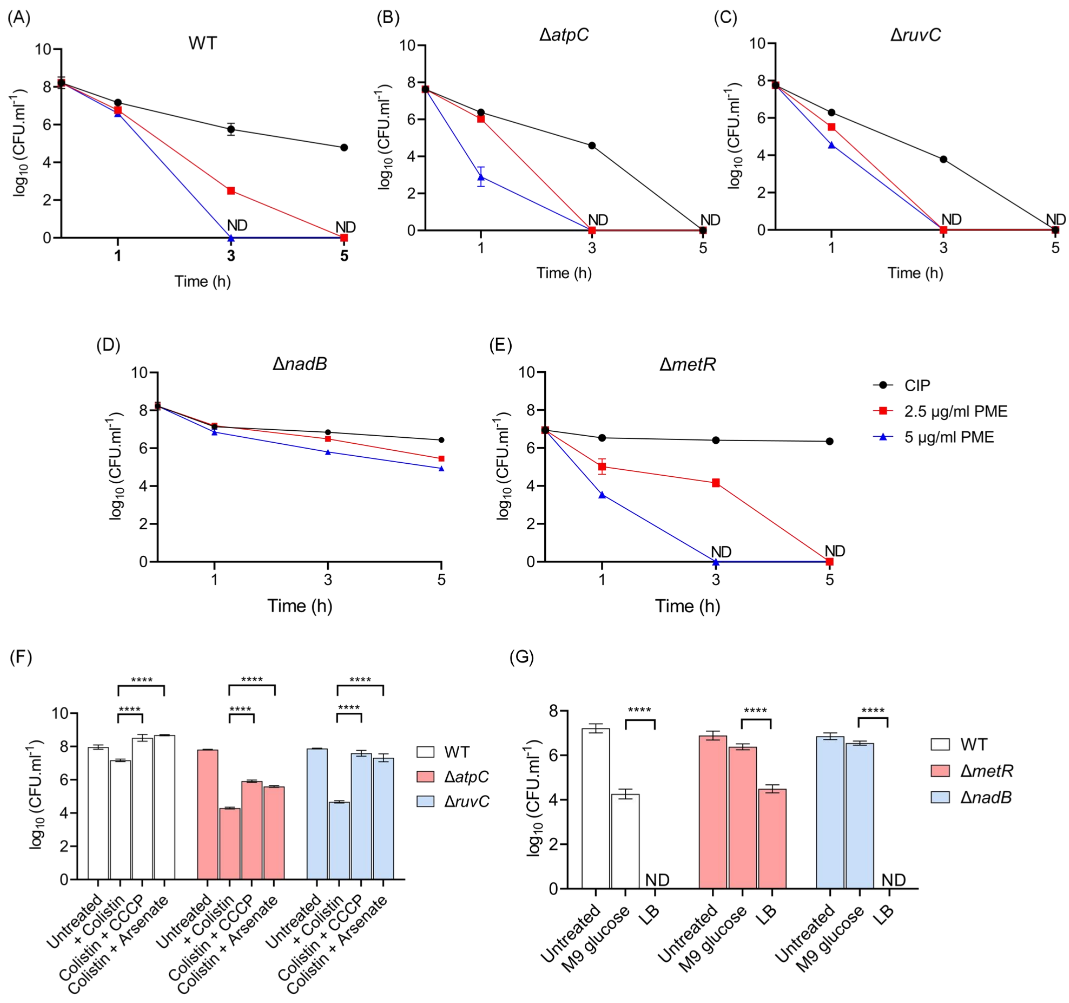

Fig. S15 Polymyxin E tolerance of CIP-sensitive and CIP-tolerant mutants

(**A, B, C, D, E**) Survival of WT (panel A), Δ*atpC* (panel B), Δ*ruvC* (panel C), Δ*nadB* (panel D) and Δ*metR* (panel E) cells following 1-h, 3-h and 5-h treatment with 2.5 µg/mL ciprofloxacin, 2.5 µg/mL or 5 µg/mL Colistin (polymyxin E, PME) during resuscitation in M9 glucose medium. (**F**) Survival of WT, Δ*atpC* and Δ*ruvC* cells following 2-h treatment with 2.5 µg/mL Colistin during resuscitation in M9 glucose medium in the presence of 100 µM CCCP or 10 mM arsenate. (**G**) Survival of WT, Δ*metR* and Δ*nadB* cells following 1.5-h treatment with 2.5 µg/mL Colistin during resuscitation in M9 glucose medium or LB medium. ND: no CFU detectable on LB dishes. All experiments were conducted in triplicate. Error bars represent standard errors. Results of analysis of variance with Tukey's HSD post hoc tests are shown; ****$p < 0.0001$.
